# A novel approach to HPV16 VLP construction and production with improved accessibility and particle sorting potential

**DOI:** 10.3389/fmmed.2026.1941186

**Published:** 2026-09-17

**Authors:** Morgan Williamson, Caleb Boren, Rahul Kumar, Lauren Gollahon

**Affiliations:** Department of Biological Sciences, Texas Tech University, Lubbock, TX, United States

**Keywords:** bimolecular fluorescence complementation (BiFC), human papillomavirus (HPV) 16, mesoscale nanoparticles, nanoparticle characterization, venus fluorophore, viral assembly, virus-like particles (VLPs)

## Abstract

Human papillomavirus (HPV), a member of the Papillomaviridae family, infects a broad range of animal hosts and is responsible for the majority of cervical cancers and a substantial proportion of oropharyngeal cancers. Current treatment strategies for HPV-associated cervical disease depend largely on disease stage, with preinvasive and early invasive lesions commonly managed through surgical excision or radiation. Additionally, more advanced cases often require chemotherapy, immunotherapy, or combination regimens. Prophylactic vaccination remains the most effective preventive approach, and currently available vaccines, including Gardasil, Gardasil-9, and Cervarix, rely on L1-based virus-like particles (VLPs) to induce protective immunity. At the same time, increasing interest has emerged in the use of nanomaterials and viral capsids as platforms for targeted drug and gene delivery, alongside the development of higher-throughput methods for nanoparticle and viral characterization. Modified HPV capsids have shown promise for cell-specific targeting and more efficient cargo delivery with reduced off-target cytotoxicity. In this study, the HPV16 structural proteins L1 and L2 were fused to the N- and C-terminal halves of the Venus fluorophore, respectively, using a specially designed linker intended to preserve capsid assembly while reducing VLP aggregation associated with current production methods. The resulting bimolecular fluorescence complementation (BiFC)-based VLPs demonstrated reduced aggregation and improved total particle yield. In addition, the particles generated a detectable YFP signal, supporting fluorescence-based monitoring of VLP assembly and providing a potential basis for high-throughput particle characterization and sorting. This intrinsic fluorescent signal may also reduce reliance on separately packaged reporter constructs for downstream analysis.

## 1 Introduction

Human papillomavirus (HPV), a member of the Papillomaviridae family, can infect a wide range of animal hosts and remains one of the most clinically significant oncogenic viruses in humans (Van Doorslaer, 2022). It has been determined that approximately 5% of all cancers worldwide can be attributed to HPV infection, accounting for 99.7% of cervical cancers, 70% of all oropharyngeal cancers, and a significant portion of other anogenital cancers (Meng et al., 2025; de Martel et al., 2017; Okunade, 2020). Structurally, HPV is a non-enveloped, icosahedral virus approximately 50–60 nm in diameter that contains an ∼8-kb double-stranded DNA genome, placing it in Group I of the Baltimore classification system (Mlynarczyk-Bonikowska and Rudnicka, 2024). The capsid is composed of about 360 L1 molecules arranged into pentamers, with 12–72 L2 molecules (de Villiers et al., 2004), or, more recently, 12–36 L2 molecules (Guan et al., 2017). HPV types are categorized by >10% differences in the L1 major capsid protein. HPV specifically targets heparan sulfate proteoglycans (HSPGs), a type of glycosaminoglycan present in the extracellular matrix, rendering most epithelial and mucosal cells permissive to infection (Cagno et al., 2019).

Cell disruption or wounding is required for both native HPV and HPV VLP infection. Because heparan sulfate proteoglycans (HSPGs) are displayed on disturbed and cancerous cells, HPV VLPs may inherently target a broad range of tumor types. One group has exploited the HPV capsids’ native ability to recognize HSPGs displayed on various tumor cell types (Kines and Schiller, 2022). This same group previously demonstrated that HPV VLPs preferentially infect tumor cells and did not enter normal cells, primary keratinocytes lacking HSPGs, or undisturbed tissue types (Kines et al., 2016). When conjugated differently, the same particle platform was shown to induce long-lasting adaptive ant-tumor immunity without the need for tumor-specific antigens (Kines et al., 2021), and to effectively treat both local and distant tumors in combination with immunotherapy (Huis In 't Veld et al., 2023). Furthermore, papilloma particles were shown to remain functional across different tumor pH and temperature conditions, which may vary with tumor density and location (Knapp et al., 2022). These studies also note that systemic delivery of HPV16 PsVs was well tolerated in mice, with no observed toxicity or significant immune effects. These works strongly suggest that HPV15 PsVs/VLPs are highly specific vectors for drug and gene therapy. With this growing evidence, continued improvement of high-yield, high-quality HPV VLP production methods will be important for supporting broader experimental and therapeutic applications.

Native HPV is not cultivatable by traditional viral culture methods *in vitro*. HPV replication is intimately linked to the differentiation of stratified squamous epithelium, and viral protein expression depends on cell cycle and immune signaling in differentiated cells. As a result, fully formed viral progeny only exist in limited amounts during natural infection. Many lines of research have therefore led to current papillomavirus production methods. Most literature using HPV16-VLPs uses the methods and recommended materials provided by Buck, Schiller, and colleagues, who developed the optimized coding sequences. These methods required the purchase of plasmids with the optimized codons under a special SV40 large T-dependent system that achieves high copy number only in 293 TT cells. The 293 TT cell line can be purchased or generated in-house by transfecting an additional SV40 large T-expressing sequence. Alternatively, coding sequences can be cloned into parent vectors under a general promoter to improve accessibility across other producer cell lines. Through related approaches, all grounded in the same core construct, critical HPV research has continued to advance. At the same time, concerns have recently been raised regarding producer cell lines that express Large T, including spontaneous packing of Large T-coding regions and the potential delivery of oncogenic DNA into healthy cells.

Understanding the native HPV capsid assembly and its co-evolution within human host systems is important for developing modified VLP models that remain efficient, reproducible, and biologically relevant. Existing VLPs used in the current vaccine platform have been L1-only and have required multivalent protection. A multivalent particle with different L1 types can assemble at equimolar concentrations to provide protection against multiple HPV types, including heterologous HPV types (Wang et al., 2020). L1 proteins assemble into pentamers that interlock primarily through disulfide bonds between Cys175 and Cys428, a conserved trait among HPV types. In addition, cryo-electron microscopy maps of HPV16 revealed numerous other L1 interactions that contribute to stable capsid formation (Guan et al., 2017). Most research studies use L1-only VLPs as they provide sufficient structural similarity for many experimental models while requiring less input material for production. Although L1 alone allows stable capsid formation, it does not fully recapitulate infectivity that seems to require L2 in native infection. L1-only VLPs are sometimes modified through the conjugation of GFP fused to a flexible Glycine/Serine linker. In host systems expressing both L1 and L2, HPV VLPs are able to assemble in multiple configurations, and native particles have been reported to contain roughly 12–36 molecules of L2. Because L1 is typically produced in excess, these systems can generate a large number of L1-only VLPs, contributing to heterogeneous particle populations, even after purification. This heterogeneity poses an obstacle to analytical techniques. In certain environments, L1-only VLPs can bind together and form larger mega-VLP structures, limiting some experimental preparations. Incorporation of L2 binding alters capsid architecture subtly, as L2 binding between L1 pentamers brings the proteins closer together and changes capsid diameter by 2–5 nm. Such slight differences are somewhat difficult to resolve without highly calibrated equipment and techniques not commonly used in cell biology settings.

Harvesting, isolating, and purifying viruses for experimental studies through ultracentrifugation-based methods requires costly equipment and supplies and carries an increased risk of particle deformation, aggregation, and contamination. Current standard approaches for assessing virus concentration, infectivity, and efficiency are often time-consuming and typically terminal for both the virus and the host cell model. The methods commonly used to determine infection efficiency, including qPCR and immunoassays, require collecting infected cells at specific time points and can become somewhat tedious and restrictive. Flow cytometry is a highly sensitive, gentle, and high-throughput method for detecting fluorophores within cell populations. With that said, its use for viral applications remains somewhat limited by a minimum detection threshold that is often above the size range of most viruses. Additionally, higher-resolution cytometers are not yet standard across all institutions. At the same time, live-cell and *in vivo* fluorescence imaging technologies continue to advance, including bimolecular fluorescent complementation (BiFC) to monitor protein-protein interactions in real time. In BiFC systems, fluorescent proteins are split into two nonfluorescent fragments, typically an N-terminal and a C-terminal half and fused to two proteins of interest. When tagged proteins interact, the fluorophore is reconstituted and emits a fluorescent signal and then stops when the interacting proteins dissociate.

The attachment of complementary BiFC fluorophore halves to the HPV16 structural proteins offers the potential to monitor productive capsid assembly in real time while distinguishing particles containing both L1 and L2 from those containing L1 alone. YFP emission generated by BiFC interaction may provide information on particle assembly, aggregation state, and intracellular trafficking, including the presence of fully assembled particles and particles undergoing disassembly or removal from the extracellular space via endocytic processes. This approach may reduce dependence on terminal sample collection and virus-specific antibody conjugates for immunocytochemistry, while also allowing the timing of VLP harvest to be assessed with relatively minimal equipment, including bright-field scopes equipped with filter inserts or filter wheels. Because the BiFC signal can be detected across multiple imaging platforms, this design may improve accessibility for construct validation and monitoring in laboratories without specialized nanoparticle instrumentation. In addition, particle populations containing both L1 and L2 may be distinguished from L1-only particles during multiple production steps. As fluorescence-based cell sorting, flow virometry, and nanoparticle detection technologies continue to advance, the BiFC signal may further support more efficient discrimination and isolation of particles containing both structural proteins, potentially reducing reliance on density gradient separation and the yield loss associated with those workflows. Intensity variation between particles may also provide a future basis for distinguishing populations with relatively high versus low L2 incorporation in higher-sensitivity flow systems.

## 2 Materials and methods

### 2.1 Plasmid construction

To detect HPV capsid protein binding or assembly/disassembly in live cells in real time using fluorescent microscopy, each protein was fused to one-half of the fluorescent protein Venus. To fuse the protein portions, various PCR techniques were used. PCR cycling conditions are reported in the supplementary (ST1), with denaturation and extension temperatures, 95 °C and 72 °C, respectively, remaining the same for all reactions. Q5 high fidelity polymerase master mix (NEB #M0492) was used for all reactions, allowing a 15 s/kB extension rate for plasmid DNA. 10 ng of template DNA, and a final concentration of 0.5 mM for each primer were added for all PCR reactions. Overlap extension PCR primers were designed for each gene and ordered for synthesis by Integrated DNA Technologies (IDT). L1 and L2 were copied from the expression vector p16sheLL (Addgene plasmid # 37320; http://n2t.net/addgene37320; RRID:Addgene_37320) (Buck et al., 2006) (Figures 1A, 2). The C-terminus half of Venus was copied from pBiFC-VC155 expression vector (Addgene plasmid # 22011; http://n2t.net/addgene:22011; RRID: Addgene_22011) (Shyu et al., 2006), and the N-terminus half of Venus was copied from pBiFC-VN173 (Addgene plasmid # 22010; http://n2t.net/addgene:22010; RRID: Addgene_22010) (Shyu et al., 2006). PCR fragments were separated on 1% agarose gel in TAE buffer. PCR fragments were extracted and purified with Qiagen Gel Extraction kit (Qiagen; #28706X4) before further cloning steps and before ligation into backbone plasmid. 2 μL (100 pg) from the ligation reaction was transformed into 40 μL of MAX Efficiency DH5*α* Competent *E. coli* cells (Invitrogen; #18258012) by heat shock. Average transformation efficiency for pBiFC-L1-VC155 and pBiFC-L2-VN173 were 7.03 × 10^9^ and 6.25 × 10^9^, respectively. Individual colonies were picked, expanded in liquid LB + AMP100 for 12–16 h, and mini-prepped using QIAprep Spin Miniprep kit (Qiagen; #27104). Promising clones of the correct size were sent to Plasmidsaurus (Eugene, OR) for sequencing.

**FIGURE 1:**
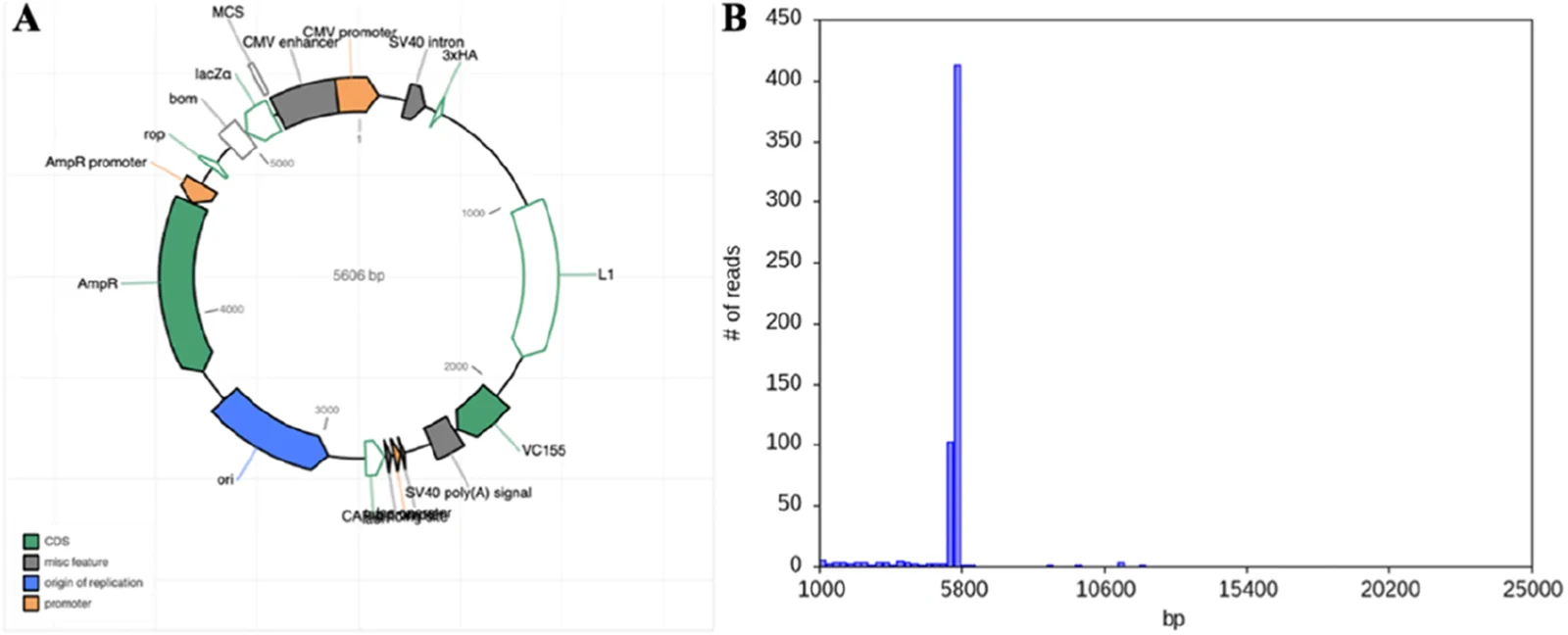
Sequencing results for pBiFC-L1-VC155. **(A)** Screenshot of the interactive plasmid map provided in the html file generated by Plasmidsaurus. **(B)** Histogram generated by Plasmidsaurus showing the size of every DNA fragment in the sample. The predominant DNA fragment was found to be the expected size, 5,606 bp.

**FIGURE 2:**
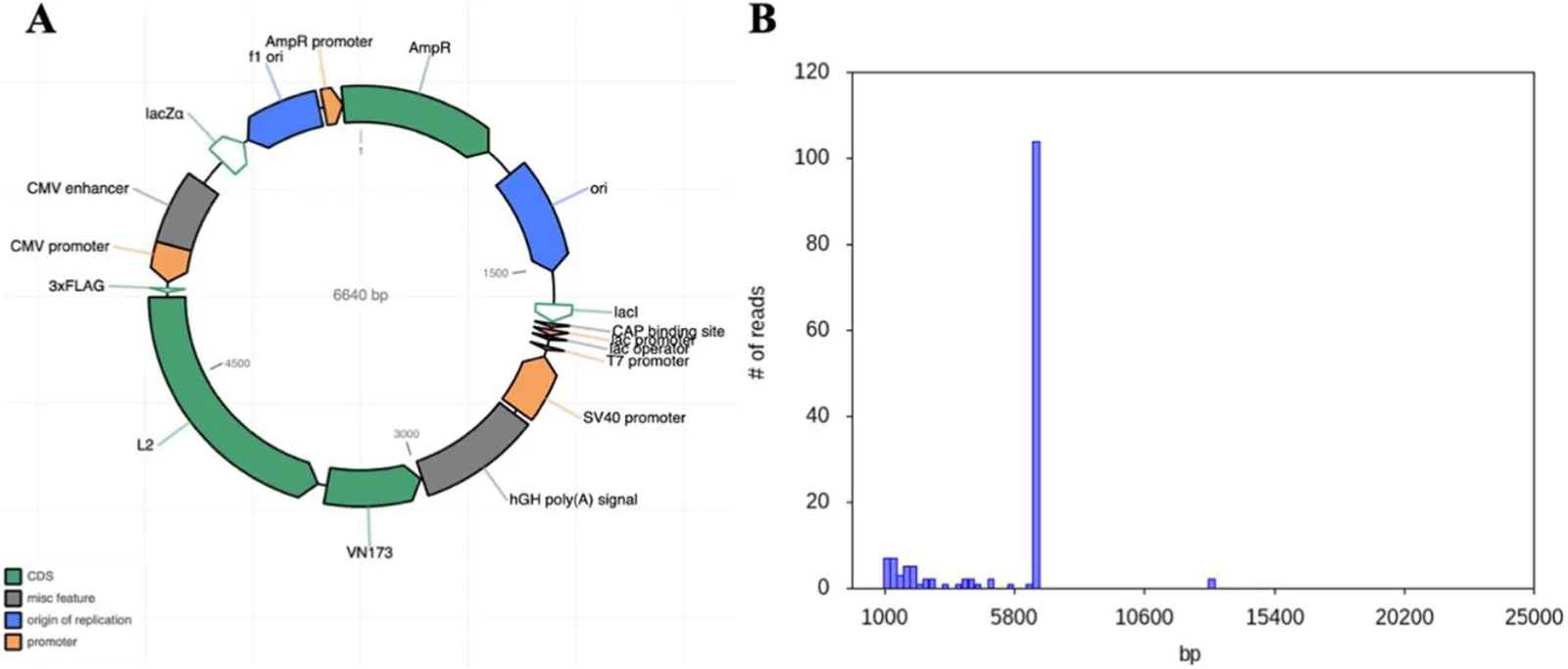
Sequencing results for pBiFC-L2-VN173. **(A)** Screenshot of the interactive plasmid map provided in the html file generated by Plasmidsaurus. **(B)** Histogram generated by Plasmidsaurus showing the size of every DNA fragment in the sample. The predominant DNA fragment was found to be the correct expected size of 6,640 bp.

### 2.2 Cell culture for VLP-producing cells

293Ts were cultured in filtered DMEM (Gibco; #12100046) containing 10% Fetal Bovine Serum (FBS) (Gibco; #12662029) and 1% penicillin/streptomycin (Gibco; #15070063). Cells were grown at 37 °C with 5% CO2 and appropriate humidity. The plasmids were transfected into 293T cells plated for ∼70% confluency after 24 h 1 h prior, cells were given fresh complete media. Plasmid DNA was complexed to LipoD293T transfection reagent in serum-free high-glucose DMEM at a ratio of 3:1 lipid:DNA. The final concentration of DNA was 0.5 μg per well in a 6-well plate or per 900 K cells. 0.5 μg of positive control p16sheLL or 0.25 μg pBiFC-L1-VC155 + 0.25 μg pBiFC-L2-VN173 was transfected per well. The lipid/DNA mixture was added to the cultures and incubated for 22 h. The transfection media was then removed; cells were rinsed with PBS and replenished with fresh complete media. 56–72 h after transfection, cells were collected accordingly for protein analysis or fluorescent imaging.

### 2.3 Immunoblotting

293T cells transfected with both plasmids were removed from the culture dish with 0.25% trypsin with EDTA (Gibco; #15050065) and pelleted by centrifugation at 4.0 × 1,000 rpm. Cell pellets were washed once with cold PBS to remove excess media and trypsin. Cell lysis for reducing assays was facilitated using RIPA buffer (Pierce) containing 0.1% SDS, 0.1% Triton X-100, and 1X HALT protease inhibitor cocktail (100X) (ThermoScientific). Total protein lysate was collected from the supernatant of the cell lysis reaction after 15 min incubation at 4 °C with mixing and centrifugation at 12 × 10,000 rpm. Protein concentration was determined from the BCA curve shown in the previous section. Samples were mixed 1:1 with 2X Laemmli Buffer + 2-BME and denatured at 95 °C for 5 min 40 μg per well was loaded onto 8%–12% SDS-PAGE gel. 5 μL of PageRuler pre-stained protein ladder (ThermoScientific; #26616). Gels ran at 130V for ∼45min. Separated proteins were semi-dry transferred (BioRad TransBlot Turbo) onto nitrocellulose membranes with transfer buffer containing 20% methanol. Membranes were blocked at 4 °C overnight with 5% non-fat milk. Primary antibodies were diluted in the blocking agent and incubated at 4 °C overnight. Anti- HPV16 L1 (CamVir1) mouse monoclonal (abcam, ab69) at a dilution of 1:2000, Anti-HPV16 L2 (2JGmab#5) mouse monoclonal (Santa Cruz Biotechnology #sc-65709) at a dilution of 1:500 in blocking buffer. The anti-E6 mouse monoclonal antibody was specific for types 16 and 18 at a 1:250 dilution. Loading control was THE β-tubulin mouse monoclonal antibody (GeneScript, #A01410), diluted 1:2,000. The primary antibody was rinsed thrice with PBST. The secondary antibody used was HRP-conjugated goat anti-mouse IgG (#A1014; Santa Cruz Biotechnology), diluted 1:2000 and added for 1–2 h at room temperature. The secondary antibody was washed off with PBST, and membranes were briefly soaked in enhanced chemiluminescent substrate (SuperSignal West Femto Maximum Sensitivity, Thermo Fisher Scientific #34095) before imaging. Blots were imaged on the Li-COR Odyssey XF Imaging System using the chemi channel and 800 channel for the ladder.

### 2.4 Immunocytochemistry

Production of VLPs in 293T producer cells and transfection efficiency were determined by fixed-cell imaging of BiFC signal and fluorescent antibody detection. 293T cells were seeded and transfected in the same manner as described in the previous section, except for the additional use of No.1.5 glass cover slips coated with Poly-D-lysine 0.1 mg/mL (Gibco; #A3890401). 48 h post-transfection, the culture media were removed, and the cells were rinsed with PBS. Cells were fixed with 4% formalin for 15 min, permeabilized for 20 min in 0.01% Triton-X, and blocked for 2 h in 3% BSA with three PBS washes between each step. Indirect immunofluorescence used the same primary antibodies as in the immunoblotting steps. Anti-HPV16 L1 (CamVir1) diluted 1:2,000, or Anti-HPV16 L2 (2JGmab#5) diluted 1:500 in 3% BSA was added to the cells and incubated overnight at 4 °C. The next morning, cells were rinsed with DPBS and incubated for 2 h with Goat anti-Mouse IgG2a conjugated to Alexa633 (Invitrogen; #A-21050) diluted 1:1,000 in 3% BSA. Cells were rinsed and immediately imaged on an Olympus BX51 or a Leica microscope using filters for FITC and Texas Red.

### 2.5 Maturation and harvest of VLPs

293T cells were removed from the culture dish with 0.25% trypsin (Gibco; #15050065) and pelleted. All media and trypsin were rinsed with PBS, and cells were pelleted again. Lysis buffer for capsid maturation made with DPBS (Gibco; #14190144) + 9.8 mM MgCl_2_. Sterile DPBS +9.8 mM MgCl_2_ was supplemented with 2.5% Ammonium sulfate buffer, 0.2% Brij-58 (ThermoFisher Scientific; #28336), 0.2% benzonase endonuclease (Millipore Sigma; #101697), 0.1% RNAse mix (Qiagen), and 100X Halt protease inhibitor cocktail (Thermo Fisher Scientific; #78429) diluted to 1X final concentration. Cell pellets were incubated in 1.4 pellet volumes of lysis buffer in a 37 °C water bath overnight. The following day, the supernatant was collected after 5 min on ice and 5 min at 5,000 x g. The remaining pellet was clarified three times with 1.2 pellet volumes of DPBS +9.8 mM MgCl_2_. The supernatant from each clarification step was pooled. A discontinuous sucrose gradient was created using 3 dilutions of a 60% w/v Optiprep™ (Iodixanol) Density Gradient Medium (MilliPore Sigma; CAS# 92339–11–2). The gradient was gently overlaid, starting with 39%, then 31%, and then 27% iodixanol in DPBS +0.85 M NaCl, in 2 mL steps. The gradient was allowed to diffuse overnight at 4 °C before the clarified supernatant was gently layered on top. Ultracentrifugation occurred at 4 °C for 8 h at 40,000 rpm in SW 41 Ti swinging bucket rotor (Beckman Coulter, #331362). Compatible thin-wall polypropylene tubes (14 × 89mm; Beckman Colter #331372) were used. A 25-gauge needle connected to a 1 mL or 3 mL sterile syringe was used to pierce the side wall of the tube at a 45° angle to collect fractions containing significant VLP content in ∼1 mL steps.

### 2.6 Electron microscopy

150-mesh copper-coated grids (Electron Microscopy Sciences, EMS150-Cu) were pre-treated with 1% alcian blue in 3% glacial acetic acid for 10 min to neutralize the negative charge. Grids were rinsed 5X and excess wicked away with filter paper. 10 mL of VLPs in Optiprep™ were allowed to attach to the grid surface for 10 min, then washed 1X in DI water. Excess liquid was wicked away with filter paper. 10 mL of L1 antibody-conjugated, 10 nm gold particles (Abcam; ab201808) was dropped onto grids for 2 min for immunogold labeled grids. Excess antibody was blotted away. Grids were negatively stained with 2% Uranyl acetate for 45 s. Grids were allowed to dry for at least 4 h before imaging on the Model H7650 Transmission Electron Microscope (TTU CASM, Hitachi) at an accelerating voltage of 100 kV.

### 2.7 Flow cytometry

The transfection efficiency of pBiFC-L1-VC155 and pBiFC-L2-VN173, and the efficient intracellular assembly of the resulting proteins in 293 T cells, were confirmed using total population flow cytometry. BiFC signal was detected using the BL1 channel on the Attune NxT Flow Cytometer (Invitrogen Catalog Number A24858). Side- and forward-scatter heights plotted the main cell population away from apoptotic bodies. Voltages used to gate the cells: FSC = 190, SSC = 290, BL1 = 300. The main population was further separated into singlets and doublets with a side scatter area vs. side scatter height density plot. Only the singlet cell population was included in the fluorescent channel counts.

### 2.8 Extraction and analysis of encapsidated DNA

HPV Pseudovirions and VLPs have been known to package plasmids of sizes similar to the wild-type genome. 20 mL of the virus sample was processed using a PCR purification kit (Qiagen; #28104) to isolate viral DNA. This method was previously described by the lab of Cellular Oncology at NIH (Cardone et al., 2014). In case the capsids could escape primary purification, 20 mL of particles were incubated in 10 mM DTT before the DNA purification step to disassemble the capsids. The resulting elution showed no bands on a 1% agarose gel. Thus, the amount of DNA was either 0 or extremely low. To rule out the second option, the eluted sample was amplified with multiple primer sets (ST1). Because the plasmids used to make the virions are around 6 kB, L1 Fwd and 155 Rev were used to assess excess pBiFC-L1-VC155 plasmid encapsidation. Primer pairs L2 fwd and 173 Rev were used to detect encapsidated pBiFC-L2-VN173. M13 universal primers were used for nonspecific DNA amplification. Finally, SV40 large T antigen primers were used to check for SV40 genetic material since most 293Ts contain integrated portions of the large T gene, which was used to immortalize the cell line originally. All primer sequences are listed in ST1.

### 2.9 Dynamic light scattering with malvern zetasizer nano ZSP

Malvern Zetasizer nano ZSP was used to measure particle size by Dynamic Light Scattering (DLS). Particle motion is related to particle size with the Stokes-Einstein equation. Measurements consisted of 10 s of light collection, repeated 9 times for each light detector. Forward and backscatter angles of 12.8° and 173° were detected on each read. The size measurement was calibrated against 10 nm beads. The 39% Optiprep™ solution was measured for background signal. The protein size setting was tested using 1 mg/mL BSA, and protein aggregation was normalized between BSA samples with or without 10 mM DTT.

### 2.10 Software and statistical analyses

All DNA components were designed and analyzed in SnapGene (version 6.2.2). All protein models were visualized in ChimeraX (version 1.5). Theoretical protein structures predicted by AlphaFold2 (Version 2.3.2) on Google Colab with AlphaColab (Mirdita et al., 2022). Fluorescent image processing and fluorescent intensity were measured in FIJI. Graphic illustration and statistical tests were performed with GraphPad Prism (v9.5.1) unless mentioned to be automatically generated from associated software.

## 3 Results

### 3.1 Sequencing of new plasmid constructs

Plasmidsaurus provided rapid, user-friendly files containing FASTA sequences, low confidence read statistics, a histogram of all DNA fragment sizes in the sample, and an. html file displaying an interactive plasmid map for each sample (Figures 1, 2).

The FASTA file containing the DNA sequence was converted to a protein sequence FASTA file using the Python package Biopython and the SeqIO and SeqIO.parse functions. AlphaColab in the Google virtual space loaded the AlphaFold2 database for predicted sequence alignments and folding predictions, showing in Figures 3, 4. There is a high degree of sequence similarity between the last two peaks, which represent the conserved linker through the L1 sequence of most HPV types (∼position 450, Figure 3) and the additional C terminus of Venus (∼position 500, Figure 4). The initial portion of the L1 sequence is also conserved across the population. Hence, the depth or number of sequences matching those regions is similar to that observed for L1 phenotypes. L1 sequence also contains regions that are specific to each type of L1, so the middle region of Figure 2 represents a low sequence similarity depth. Similarly, Figure 4 shows regions of L2 protein conserved across all HPV types, indicating greater sequence depth throughout the entire L2. The very last two peaks (positions ∼500–600, Figure 4) correspond to the N terminus of Venus, which was added with high sequence similarity.

**FIGURE 3:**
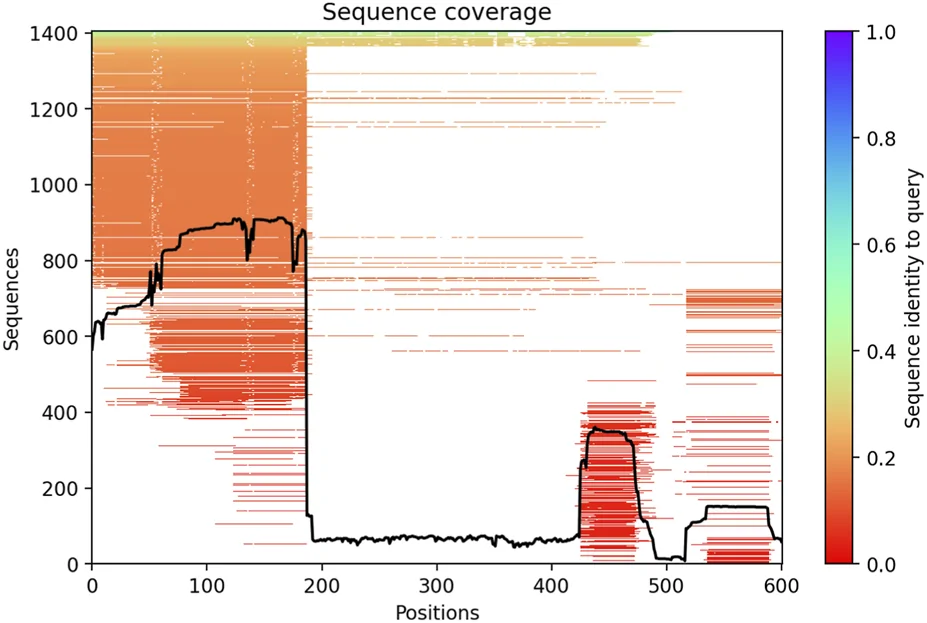
AlphaFold-generated graph of sequence similarities across database entries for the coding region of pBiFC-L1-VC155. The left-hand Y-axis represents the number of sequences from the database with similarity to the entered sequence, or the depth. The X-axis represents the residue position in the sequence of interest. The gradient color scale bar indicates the degree of sequence similarity to the entered sequence, with values close to 0.0 considered significant.

**FIGURE 4:**
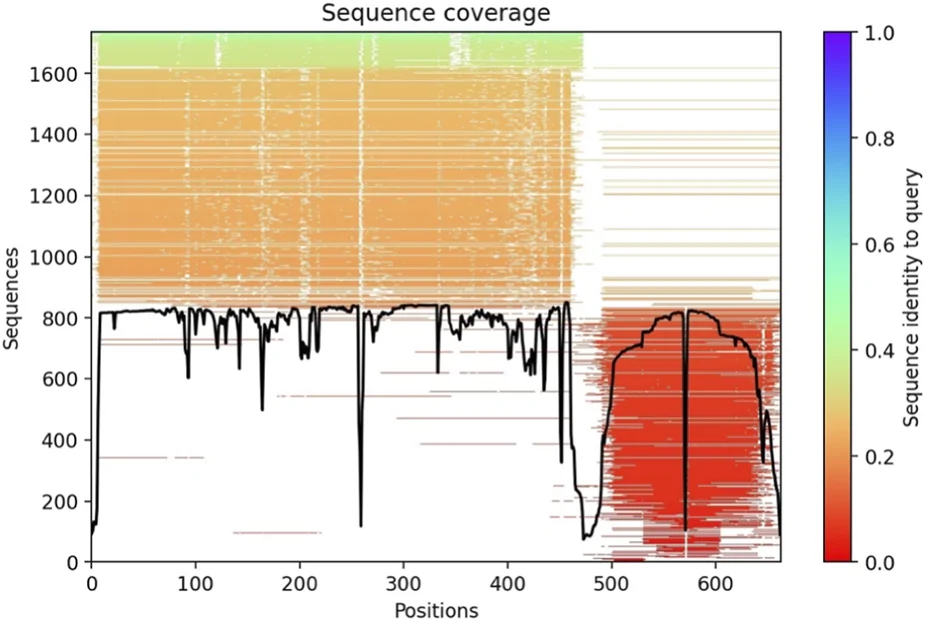
AlphaFold-generated graph of sequence similarities across database entries for the coding region of pBiFC-L2-VN173. The Y-axis shows the number of database sequences that are similar to the entered sequence. The X-axis represents the residue position. The gradient color scale bar indicates the degree of sequence similarity to the entered sequence, with values near 0.0 considered significant.

### 3.2 Estimation of protein properties for L1-VC155 and L2-VN173

*In silico* modeling of translation from the resulting plasmids showed theoretical protein properties, including absorbance, extinction coefficient, and molecular weight (Table 1). Proper theoretical modeling was necessary to reduce trial-and-error in method development by providing sufficiently close estimates of the various calibrations/settings required before a software or measurement instrument can be used accurately. These values also helped set up appropriately controlled experiments. Measured characteristics of the actual protein products can be compared to the theoretical characteristics to validate the design and characterization approaches.

**TABLE 1:** Theoretical properties of L1-VC155 and L2-VN173 proteins.

Property	L1-VC155	L2-VN173
Length (aa)	601	663
Molecular weight (Da)	67,842.72	72,035.25
Extinction coefficient at 280 nm (M^-1^ cm^-1^)	48,360	50,200
Absorbance (280nm, 0.1%)	0.71	0.70
Isoelectric point	10.26	6.53
Charge at pH 7	35.72	−5.63

Given the predicted molecular weight for each protein, an estimated molecular weight range was calculated for comparison with the actual size. This was determined by the following calculation: 72 pentamers (72 × 5) = the total number of L1 subunits expected per capsid.

$$\text{Weight of total }L1=\left(360\right)\;x\;67.84272\text{ kDa}=24,423.379\text{ kDa}$$

Because the number of total L2 subunits per capsid is variable, upper and lower limits for the weight range were determined from a simple linear equation:

$$\text{Total capsid weight }\left(\text{kDa}\right)=72.03525\left(N\right)+24,423.379$$

*where N can be substituted for any number between 12 and 36, dependent upon the reported L2 copy number. The ranges representing the total weights of 12 and 36 L2 subunits are 864.423 kDa and 2,593.269 kDa, respectively. Thus, the estimated range for the total capsid (dry) weight is 25,287.802–27,016.648.

The slight increase in expected mass could lead to differences in diameter, density gradient migration, 2D image deformation, surface charges, and the overall icosahedral pattern. The fasta sequences were uploaded to AlphaFold for tertiary and quaternary structure predictions. The resulting PDB files were visualized in ChimeraX as shown in Figure 5.

**FIGURE 5:**
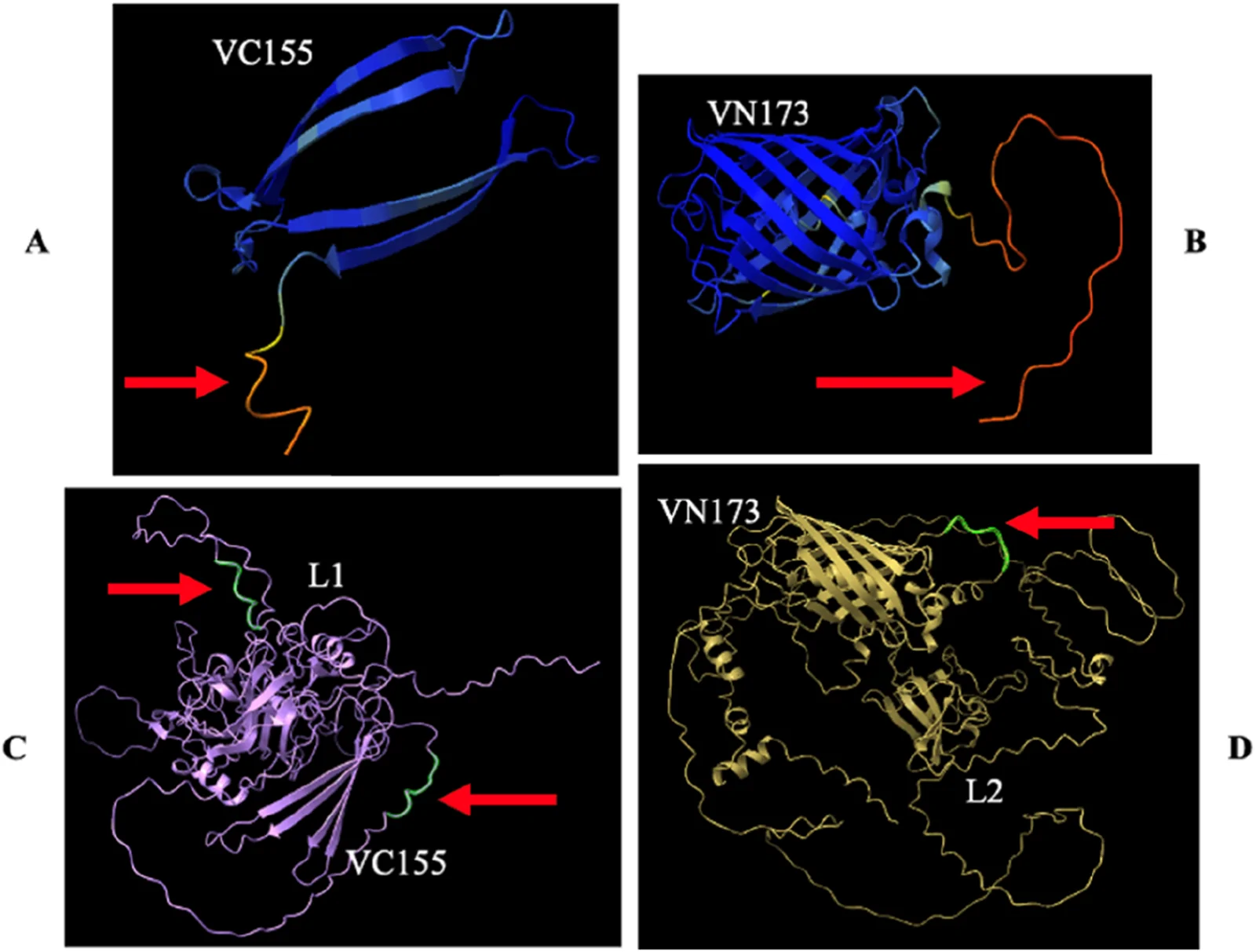
Predicted folding for L1-VC155 and L2-VN173. **(A)** AlphaFold high confidence fold of VC155 with linker added. **(B)** AlphaFold high confidence fold of VN173 with linker added. **(C)** AlphaFold prediction of L1 fused to VC155, where the linker sequences appear in one natural location (arrow), and one added location near VC155. **(D)** AlphaFold prediction of L2 fused to VN173, where the linker sequence only appears in the added location near VN173 (arrow).

Per-residue confidence (pLDDT) was estimated on a scale of 0–100 and plotted in correlation to lDDT-Ca metric (Mirdita et al., 2022; Mariani et al., 2013). Very confident (100 ≥ pLDDT ≥90) or confident (90 ≥ pLDDT ≥70) portions of the sequence were observed, giving overall confidence to the local structure within each domain (Figure 6A). The Predicted aligned error output showed expected positional error at residue *X,* if predicted and actual structures (Ca, N, and C atoms) are aligned on *Y*. A low score for any two residues (*X, Y*) in different domains indicates confidence in the position of each domain in relation to the other. Confidence is reflected in pixel intensity, and each domain with significant confidence is color-coded in the visualization (Figures 6B,C).

**FIGURE 6:**
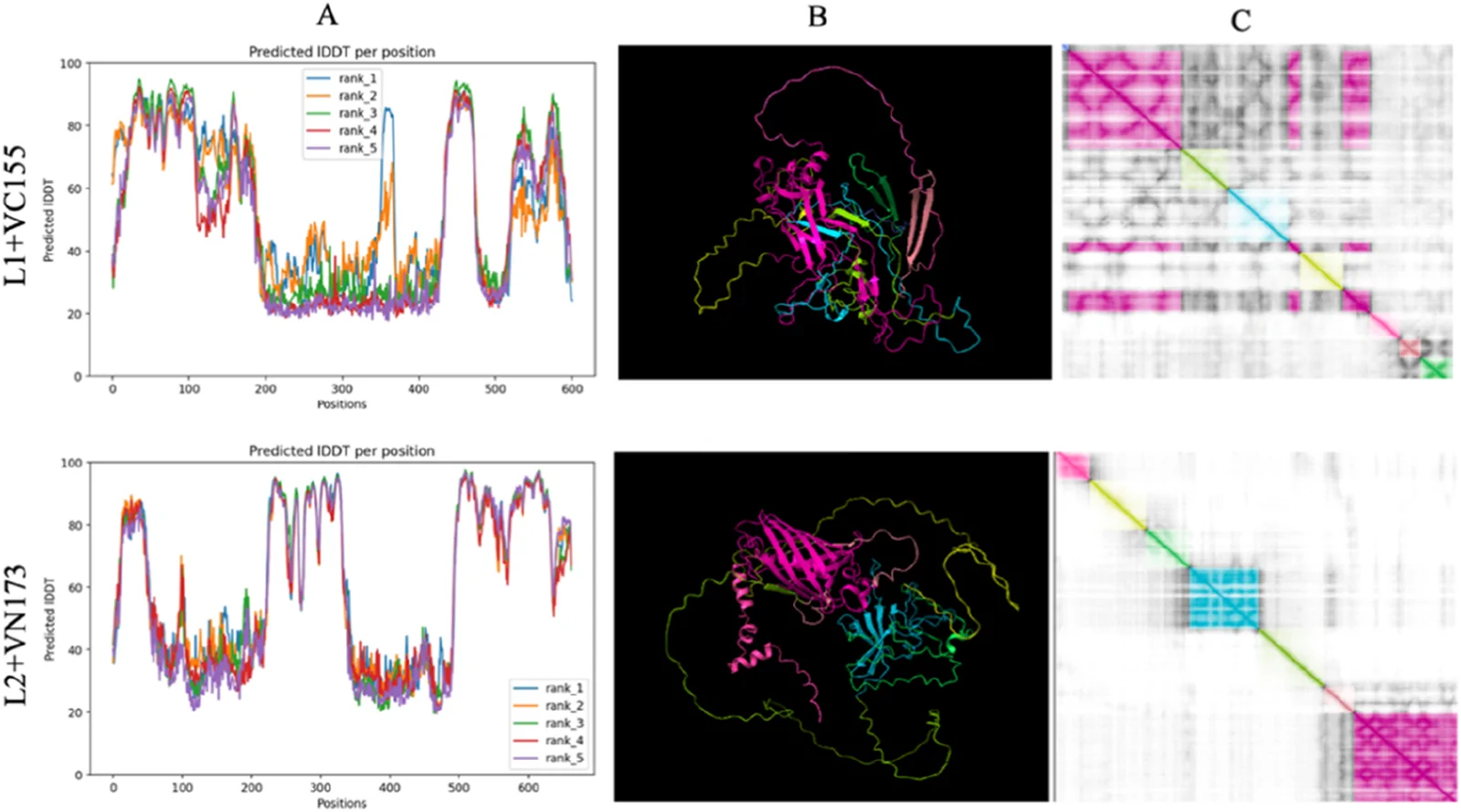
Confidence measures pLDDT and PAE for predicted structures. **(A)** pLDDT confidence by residue position per prediction. **(B)** Visualization of each domain in the same colors as the PAE. **(C)** Structure alignment error with high confidence in colored portions.

Fusing the proteins to the HPPAPKED linker appears to allow very close proximity between the fluorophore halves without straining the L1 or L2 conformation or obstructing the tertiary or quaternary structures involved in various capsid-binding mechanisms (Figure 7).

**FIGURE 7:**
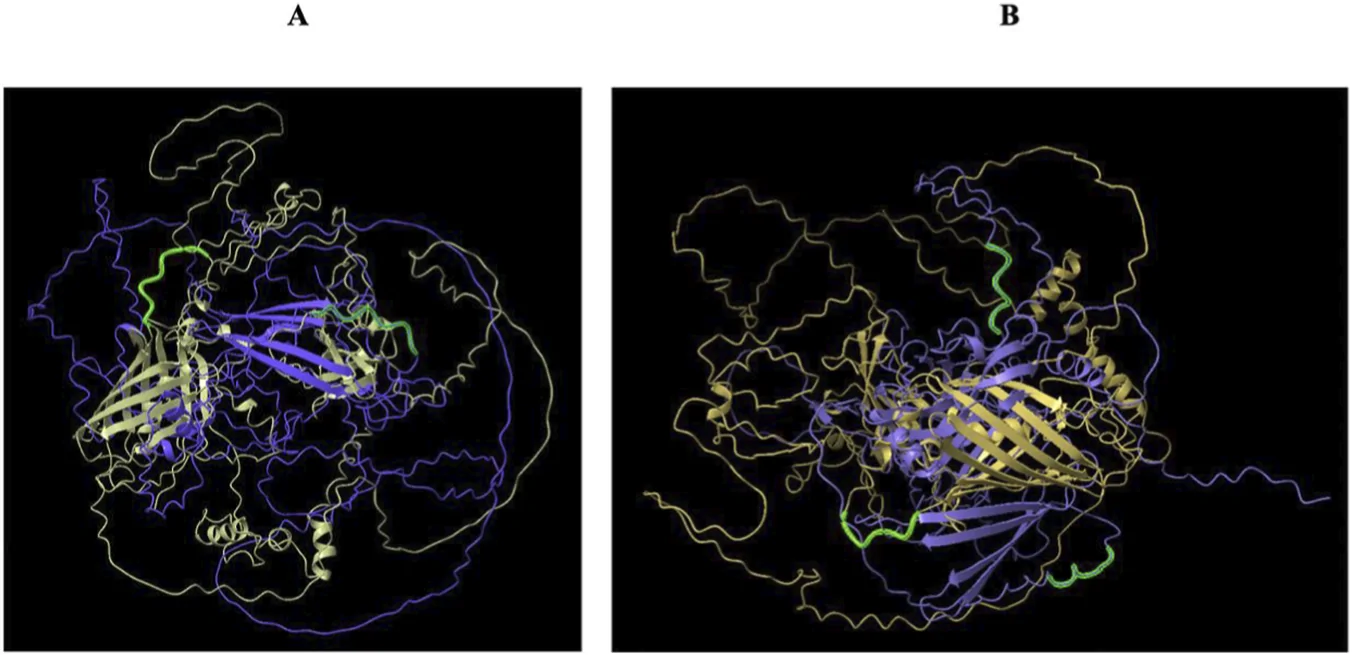
Predicted folding and interaction between a single peptide of both L1-VC155 and L2-VN173. **(A**,**B)** Represent two different visualization angles of both peptides and their predicted association. The linker sequence is highlighted in green.

### 3.3 Expression of recombinant proteins in 293T producer cells

The total lysate in reducing buffer, from 293T cells transfected with p16sheLL, was expected to show some L2. However, since the proteins are co-expressed under a single promoter and for the reasons described above, L1 was expected to be more abundant. 293T cells transfected with pBiFC-L1-VC155 and pBiFC-L2-173 were expected to have relatively equal amounts of L1 and L2 in the total reduced lysate since each gene is under a separate and identically strong promoter (CMV).

Results from SDS-PAGE-separated immunoblots mostly matched the expected weights from Table 1. The negative control sample that was transfected with pBiFC-VC155 and pBiFC-VN173 showed no expression of either L1 or L2. Cell lysates transfected with the positive control p16sheLL showed moderate L1 expression around 55 kDa, as expected and reported by other groups (Buck et al., 2004). The same positive control sample did not show L2 expression, which was surprising. The expression efficiency of p16sheLL was expected to be slightly lower overall. Due to differences in large T antigen expression in the host cell, the amount of L2 produced may be below the limit of detection for immunoblotting. Alternatively, since the binding site of L2 is often hidden in mature capsids, little to no L2 signal is expected if incomplete disassembly of already folded and assembled L1:L2 complexes or immature capsids.

293T cells transfected with pBiFC-L1-VC155 and pBiFC-L2-VN173 showed strong expression of L1 around 60 kDa, as predicted in the previous section. The same population showed L2 expression at approximately 70 kDa, as predicted by the initial modeling. The loading control b–Tubulin was detected in all three lysates around 50 kDa with equal loading (Figure 8).

**FIGURE 8:**
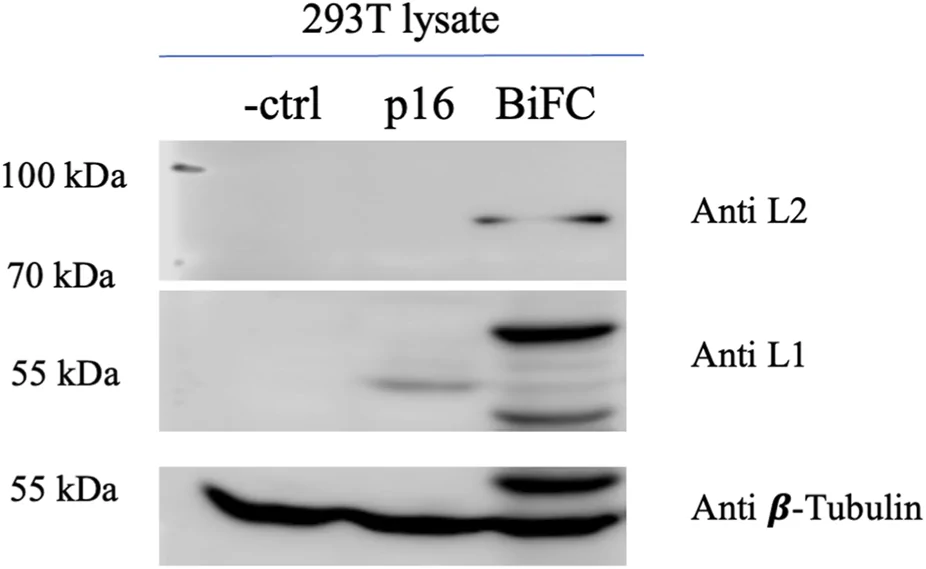
Immunoblot of 293T cell lysates transfected with p16sheLL or pBiFC-L1-VC155/pBiFC-L2-VN173 or control non-transfected. 40 mg of total cell lysate mixed 1:1 with Laemmli buffer + 2-BME was loaded per well. Anti-HPV16 L1 (CamVir1), Anti-HPV16 L2 (2JGmab#5), and Anti-b-tubulin (GeneScript) were imaged using the Li-COR Odyssey XF Imaging System.

Relative protein expression in blots was extrapolated from Fiji-generated intensity values. The rectangular selection tool was used to select each band, using the same Region of Interest (ROI) for each sample probed with the same protein. The mean greyscale value was recorded for each band; this represents the mean pixel density within the rectangular selection. Each band intensity was normalized against a background intensity taken from the same lane. Significant expression differences were determined by comparing volumetric signal intensities with those of the loading control bands. The average across all replicates is displayed in Figure 9.

**FIGURE 9:**
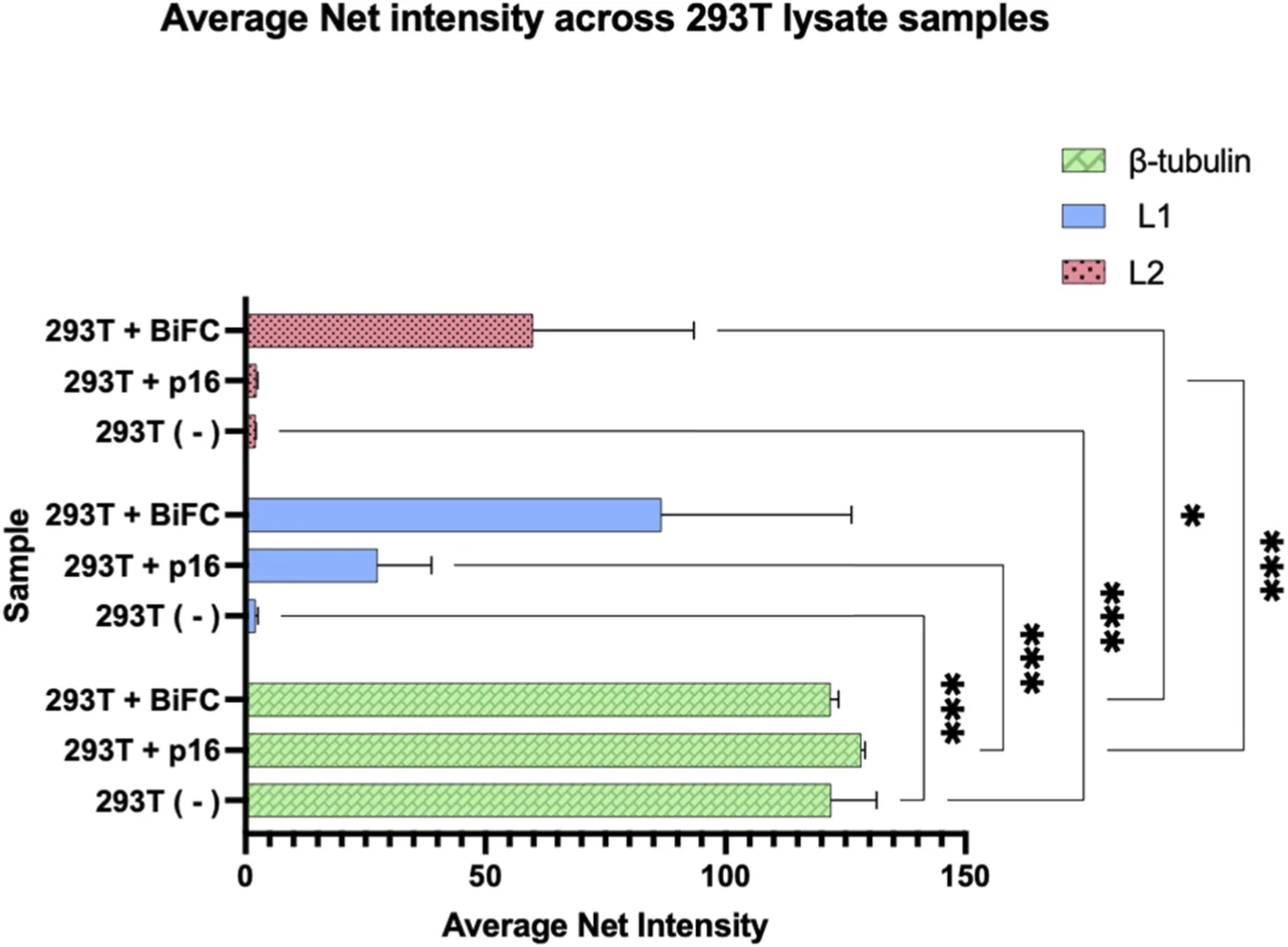
Relative intensity of antibody-HRP signal for plasmid constructs expressed in 293T cell lysates. Significant differences in expression between each sample within its group are marked by * for p < 0.05 and *** for p < 0.001, after Bonferroni correction for multiple comparisons.

A two-way ANOVA with Bonferroni multiple comparisons correction was conducted between each group and between each sample within the group. Between L1, L2, and β-tub groups, there were significant differences in intensity/expression (F (2, 9) = 11.54). The negative control group (293T (−)) had no expression of L1 or L2. In comparison, the positive controls (293T + p16 and 293T + BiFC) showed some expression, with significant differences between the negative control and the other 2 groups (Bonferroni-adjusted p < 0.005 for each group comparison). Expression levels between the loading control, L1, and L2 were expected to differ following transient transfection. There was no significant difference among samples within the loading control group, confirming, as expected, uniform expression between samples derived from the same 293T cell stocks, which should have similar housekeeping gene expression. L1 expression was most significantly different between the negative control and the BiFC-transfected samples (p < 0.005). p16sheLL-transfected samples also showed significantly different intensities between untreated samples (p < 0.05). L2 intensity was significantly different between the negative control and BiFC-transfected samples (p < 0.05), but there was no significant difference between the negative control and p16sheLL-transfected samples.

### 3.4 Transfection efficiency

Transfection efficiencies for each sample were normalized against an unstained control (UC) of 293T cells transfected with unmodified parent vectors, pBiFC-VC155 and pBiFC-VN173. Averages for each time point were calculated across 3 technical and 3 biological replicates. Figures 10–12 show a representative average across one set of technical replicates for each collection point. The peak BiFC signal intensity across 3 biological replicates was 17.6165% at the 72-h time point, the adjusted mean. The 96-h time point consistently showed more cells in the very bottom-left corner of the density plot (FSC-H vs. SSC-H), consistent with the appearance of cells at collection time. 293T cells were lysed for capsid particle collection at 72 h post-transfection based on peak BiFC intensity and effects of cell health on yield.

**FIGURE 10:**
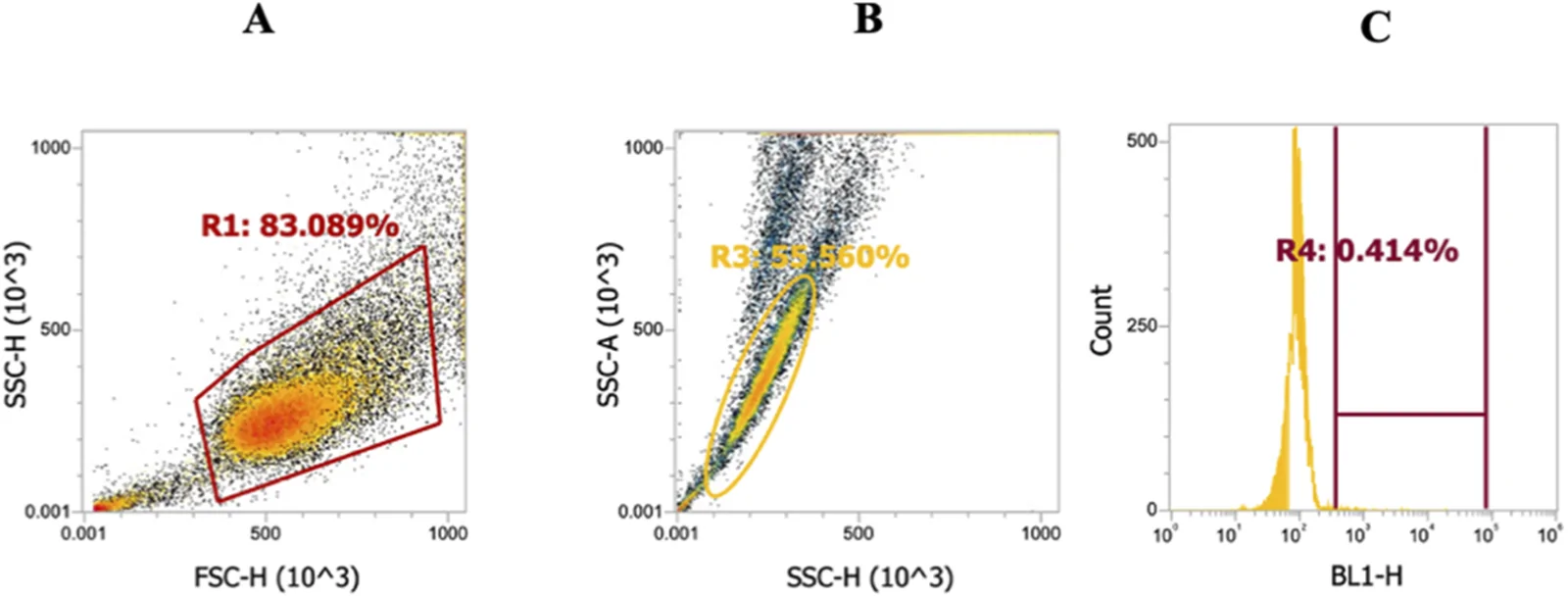
Flow analysis of the unstained control. **(A)** Represents the separation of the cell population by size gating. **(B)** Represents the gating for singlet cells from the population in **(A)**. **(C)** Represents the percentage of the population in **(B)** that are positive for YFP emission, measured using the BL1 channel on the Attune NxT flow cytometer.

**FIGURE 11:**
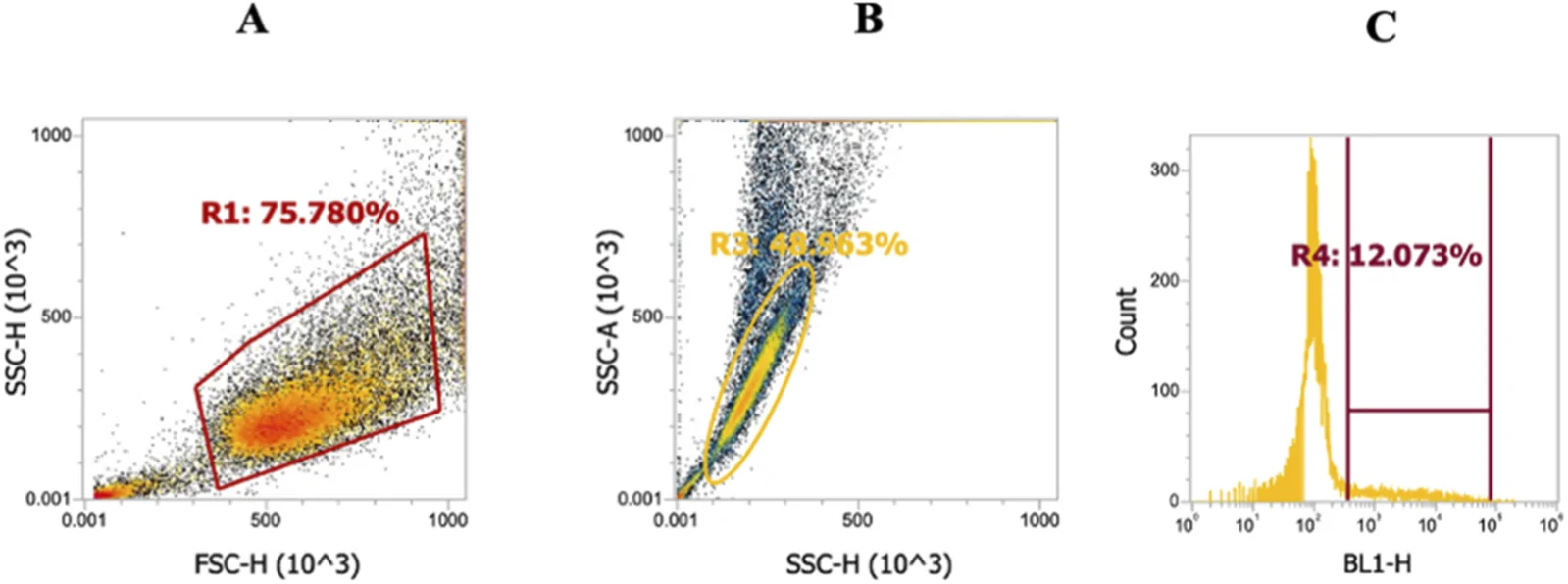
Flow analysis of 293T cells transfected with pBiFC-L1-VC155 and pBiFC-L2-VN173 after 48 h. **(A)** Represents the separation of the cell population by size gating. **(B)** Represents the gating for singlet cells from the population in **(A)**. **(C)** Represents the percentage of the population in **(B)** that are positive for YFP emission measured using the BL1 channel on the Attune NxT flow cytometer.

**FIGURE 12:**
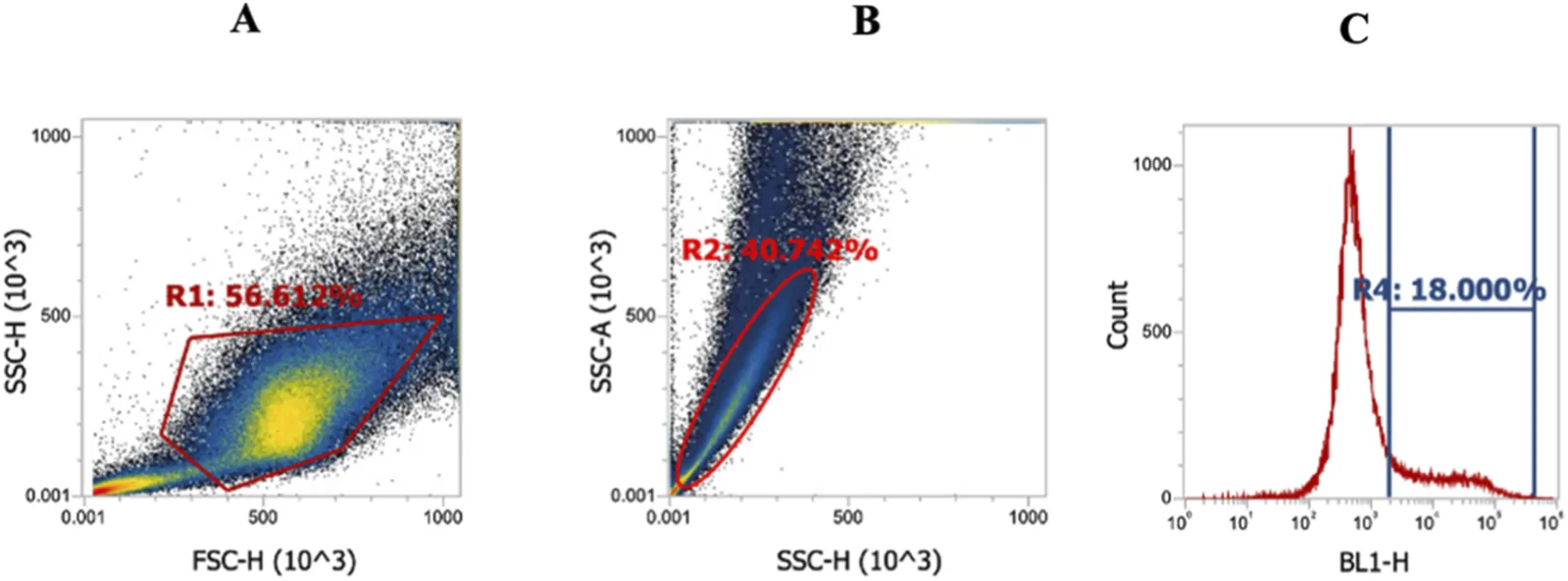
Flow analysis of 293T cells transfected with pBiFC-L1 VC155 and pBiFC-L2-VN173 after 96 h. **(A)** Represents the separation of the cell population by size gating. **(B)** Represents the gating for singlet cells from the population in **(A)**. **(C)** Represents the percentage of the population in **(B)** that are positive for YFP emission, measured using the BL1 channel on the Attune NxT flow cytometer.

The ease of monitoring transfection efficiency and efficient intracellular assembly with BiFC signal was assessed with live and fixed cell fluorescent imaging on multiple types of fluorescent scopes with varied filter sets and total magnifications, including upright epi-fluorescent Olympus BX-51 (Figure 13), inverted epi-fluorescent Leica microscope (Figure 14), and Nikon Ti-2 Eclipse live cell compatible inverted confocal microscope with LED light excitation (Figure 15). YFP emission is imaged in transfected 293T cells successfully expressing both plasmids and displaying efficient intracellular assembly of VLPs. Live-cell/real-time imaging of BiFC-producing capsids revealed swollen cells, another visual indicator of VLP production in 293T cells (Buck et al., 2004).

**FIGURE 13:**
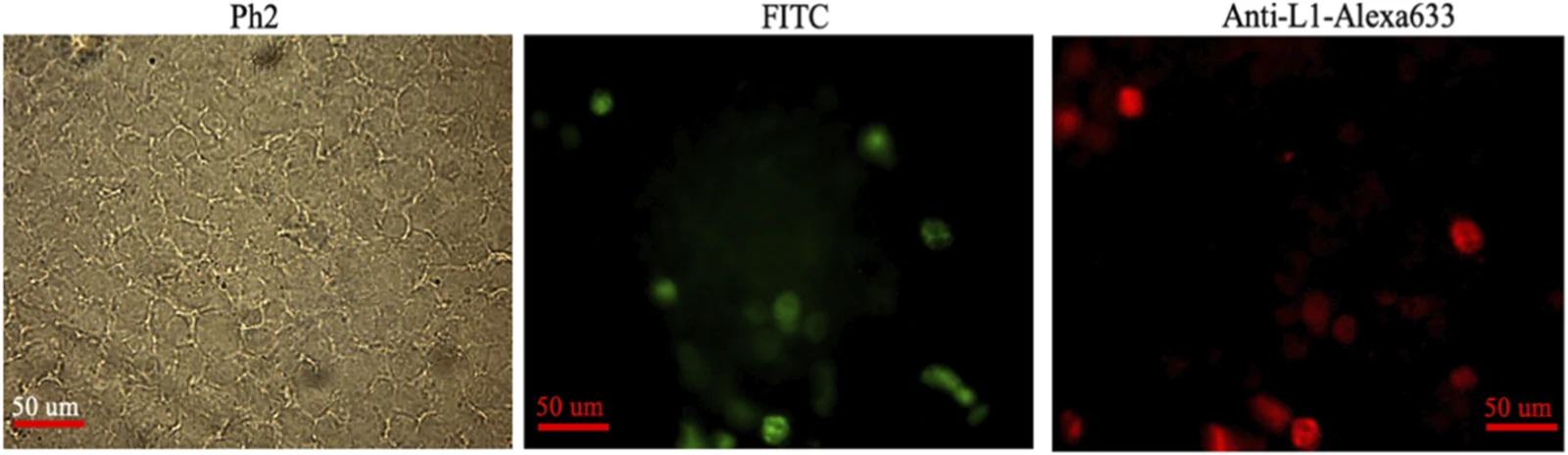
Fluorescent imaging of BiFC and L1 antibody binding in fixed 293Ts on BX-51 (Olympus) with filters FITC and PI. Images were taken with a ×40 objective and a total magnification of ×400 using the BX-51 associated software and DP controller (Olympus). BiFC signal from correct capsid assembly before cell fixation was viewed with the FITC filter. Cells were stained with anti-L1, then with a secondary antibody (Alexa 633-conjugated goat anti-mouse), and imaged in the PI channel.

**FIGURE 14:**
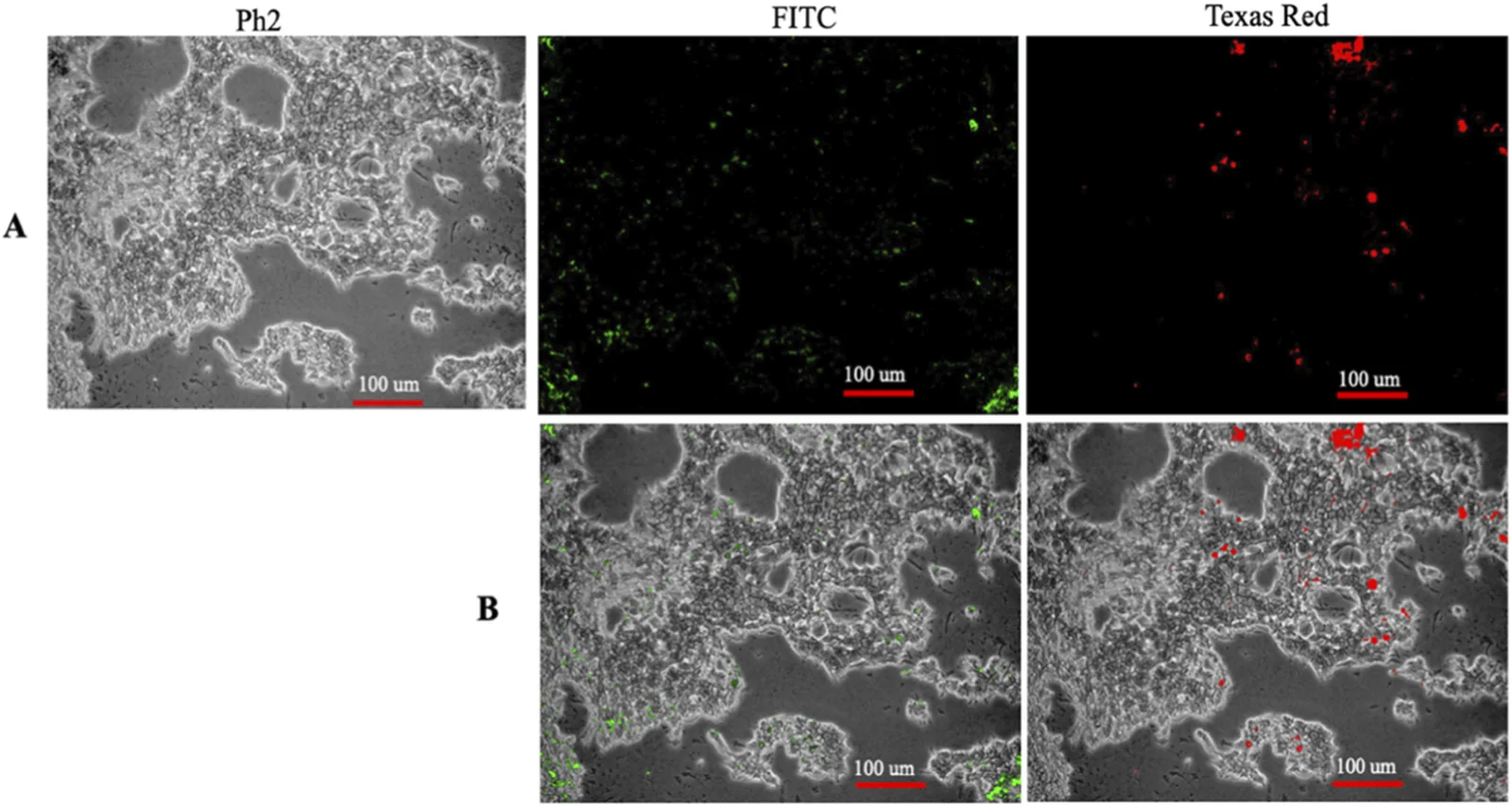
Fluorescent imaging of BiFC and L1 antibody in fixed 293Ts on Leica with filters FITC and Texas Red. Images were taken at a total magnification of 200X with a ×20 objective, and the scale bar represents 100 microns. Row **(A)** shows each channel image separately, with each column labeled by the filter used. Row **(B)** is the merge of each fluorescent channel with the phase contrast channel.

**FIGURE 15:**
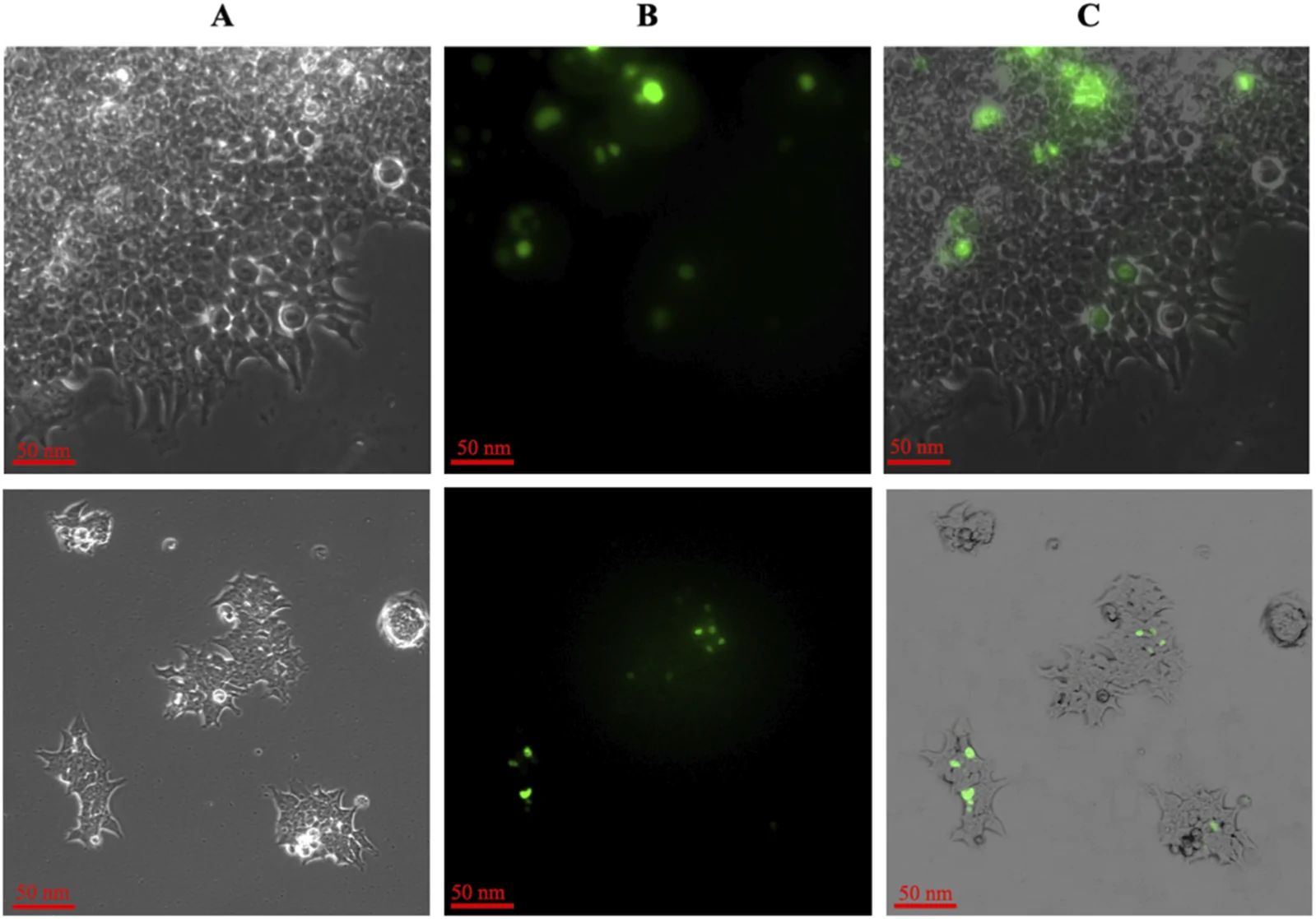
Fluorescent imaging of BiFC in live 293T cells on Nikon Eclipse Ti 2 (Nikon). Images were taken in real time at a total magnification of ×400 using a ×40 objective and Ph2 **(A)** and YFP **(B)** filters. Scale bar represents 50 microns. Phase and YFP channels were overlaid in Fiji **(C)**.

The relative intensity of the BiFC signal is not amplified and reflects the actual number of capsids emitting YFP. L1 antibody intensity is much brighter as it is amplified with the indirect immunofluorescent technique. Because of differences in peak intensities, merging all three fluorescent channels in most images (regardless of scope type) obscured much of the BiFC signal, even with increased opacity of the red channels.

X- and Y-coordinate matching in both green and red channels with Fiji enabled accurate counting of cells producing L1/L2 capsids. The intensity measurement of a neighboring cell without a signal was subtracted from the intensity measured of the cell with a signal across both channels. Each selection measured had the same pixel area included in the mean grey value calculation. If the cell had significant intensity relative to the background signal in both channels, it was counted as having both plasmids and L1/L2-containing capsids, and its signal intensities were added to calculate the average signal intensities. If only significant intensity were measured in the red channels, those cells were counted as L1-only capsids and did not take up pBiFC-L2-VN173 during transfection. Those cells that took up no plasmids or only pBiFC-L2-VN173 were grouped (No signal) by subtracting the other categories from the total population. Figure 16 demonstrates each category stacked in a bar, representing the total population in the image sample.

**FIGURE 16:**
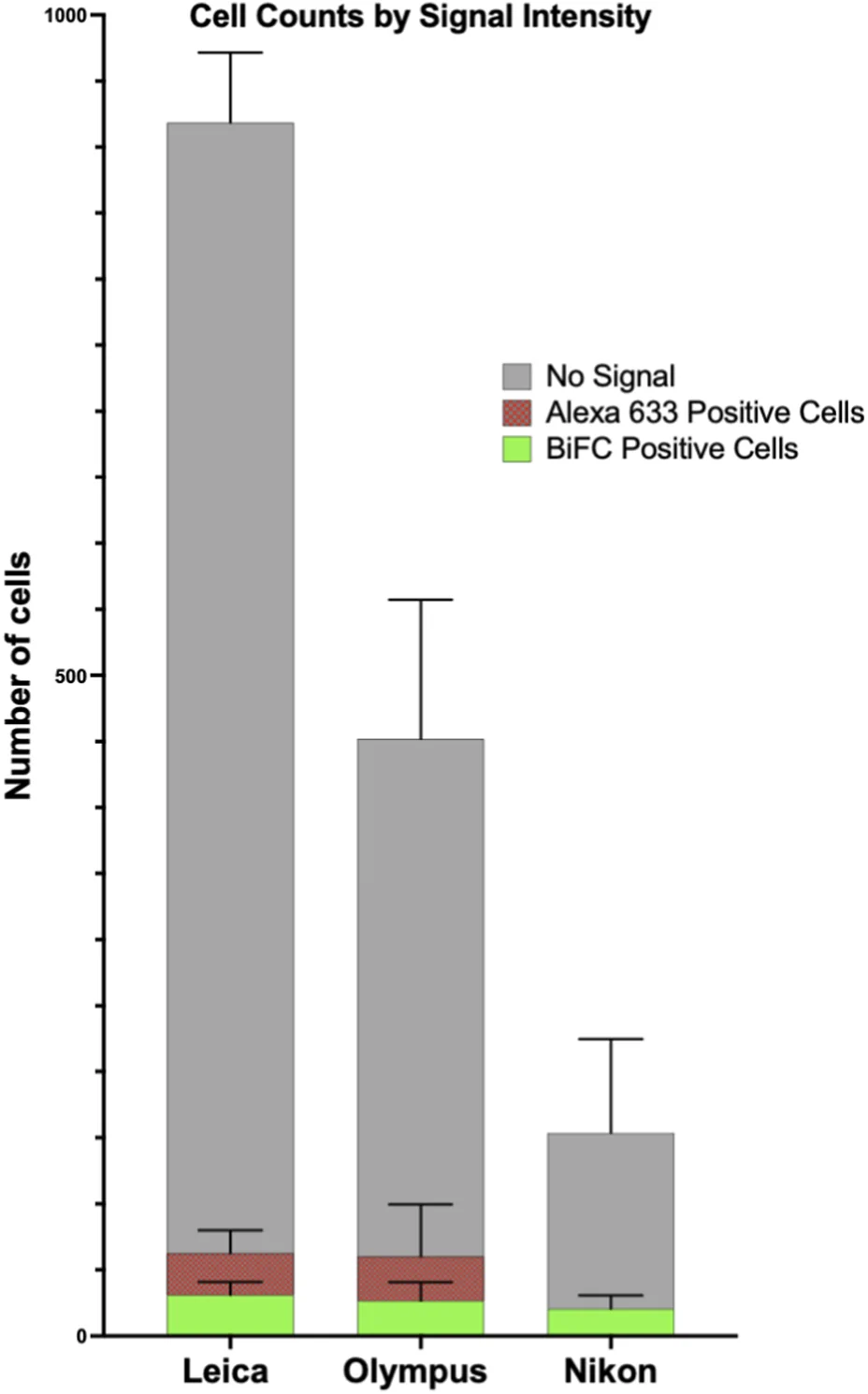
Number of BiFC-transfected 293T cells with significant intensity in the red channel only or both red and green channels. Green/Yellow and Red channels imaged on various fluorescent microscopes were loaded in Fiji and gated for intensity before counting.

As expected, the percentage of L1-only producing cells is higher than the percentage of L1/L2 producing cells in each scope type/filter set tested. For all fixed imaging, regardless of filter set, the BiFC signal was detected in about half the cells shown in the anti-L1-Alexa 633 channel. The standard error in each group is most likely due to slight variation in imaging time post-transfection with BiFC plasmids or in fixation/immunofluorescent processing across samples. Differences in total magnification, excitation and emission filters, and light source between scopes can account for differences in green/red detection and the number of cells per field of view between scope types. Fixed-cell imaging of Alexa 633-stained L1 antibody confirmed that these cells were successfully producing HPV16 capsid proteins, and the cells’ increased size indicated efficient assembly into capsids that can withstand intracellular degradation attempts for up to 96 h; longer times were not imaged. Live-cell and real-time imaging did not allow for the terminal antibody-binding assay in the Nikon scope group. The stage of the Nikon scope was equipped with a fully functional incubation chamber and held only certain types of culture plates that allowed the appropriate working distance for the objectives.

### 3.5 Particle isolation, yield, and purity

Using 2 mL steps for each of the three OptiPreps, created a sedimentation path that filled approximately half the tube length, averaging 43.2 mm and showing no significant variation between layers. Figures 3–18 displays an example of a diffused gradient, with clarified lysate layered on top, prior to ultracentrifugation (Figure 17A). DPBS +9.8 mM MgCl_2_ was used to clarify lysed cell material and to fill the remaining volume of the ultracentrifuge tube to avoid crushing (Figure 17B).

**FIGURE 17:**
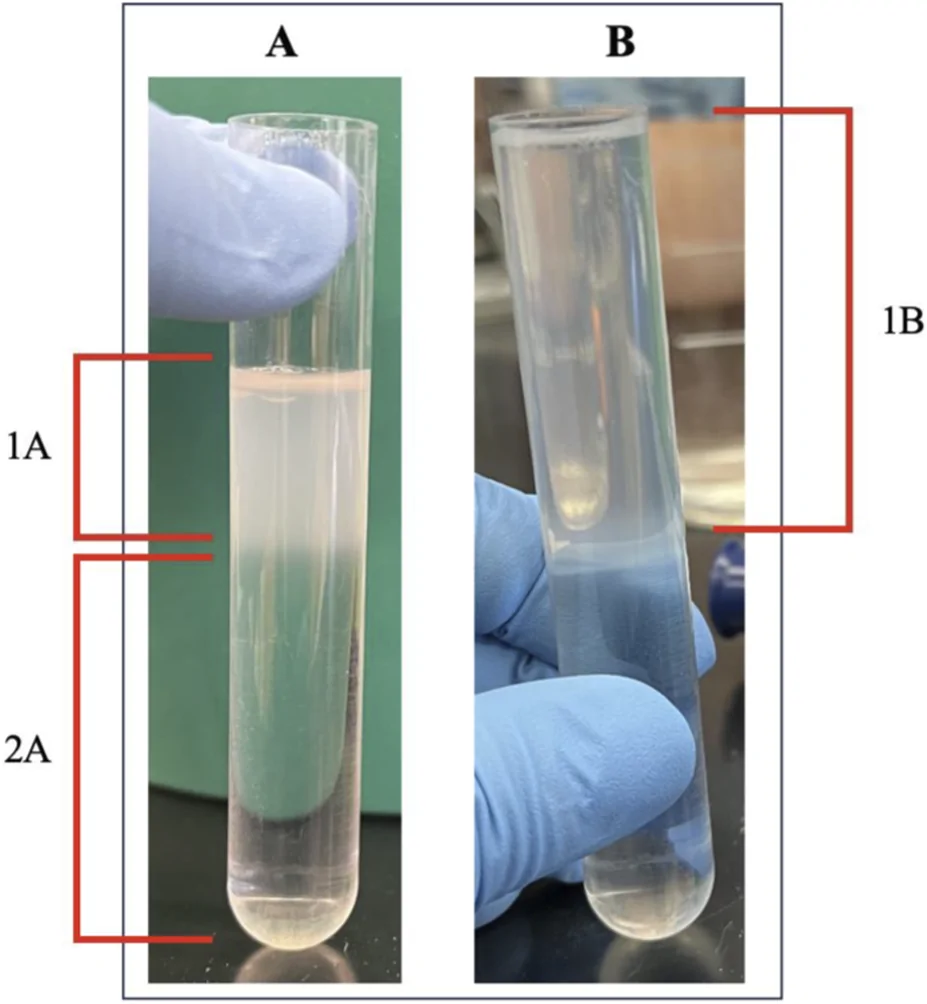
Gradient volume and sample volumes layered before ultracentrifugation. **(A)** Total clarified lysate loaded onto the gradient is within bracket 1 A. The sedimentation path, or entire length of the OptiPrep gradient, is within bracket 2 A. **(B)** 1 B depicts the final volume added to the tube before ultracentrifugation. The clarified and pooled lysate was diluted with additional DPBS+9.5 mM MgCl2 to achieve the recommended fill height and prevent crushing.

**FIGURE 18:**
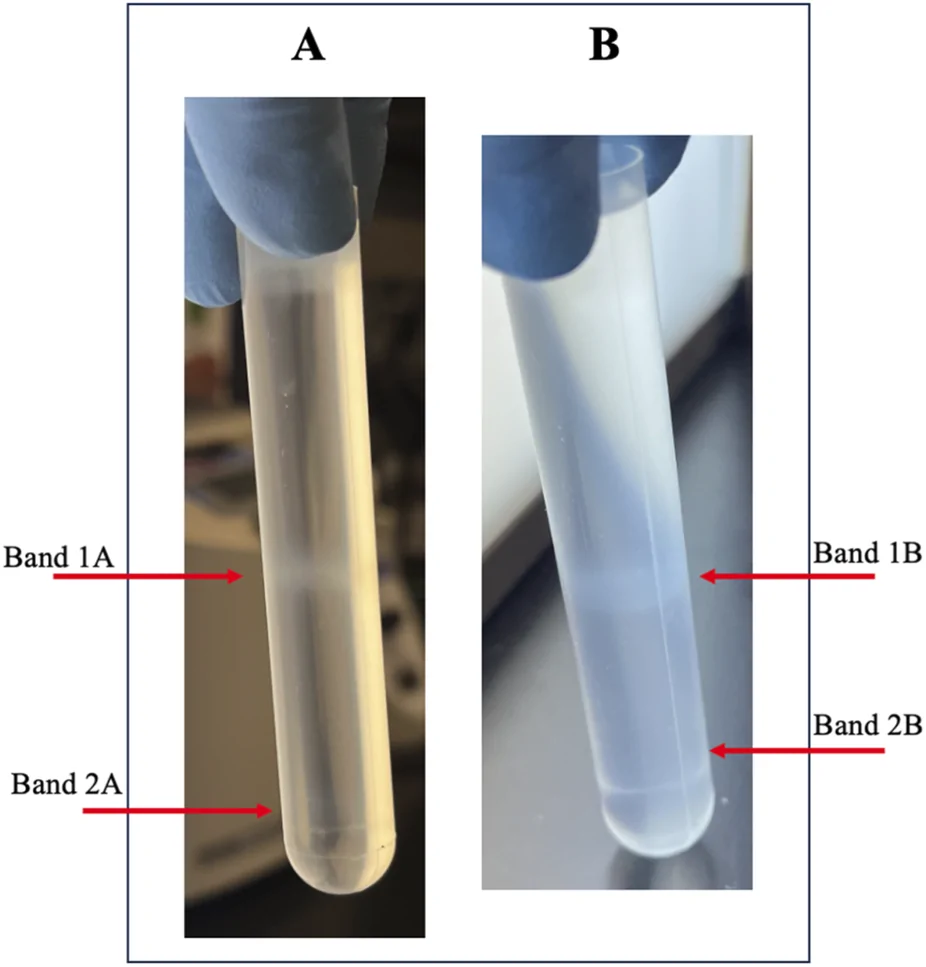
Separation of clarified lysate from 293T cells expressing **(A)** p16sheLL, **(B)** pBiFC-L1-VC155, and pBiFC-L2-VN173. Band 1 represents the interface between the top layer of Iodixanol and the clarified lysate components of low density. Band 2 represents the accumulation of virus-like particles produced by each construct.

P16sheLL density is reported to form a band around a third of the sedimentation path, measured up from the bottom, placing the particles between the 33% and 39% layers with an average particle density of 1.25 g/mL. Positive control p16sheLL produced a grey band at approximately ¼ of the way up from the bottom (Figure 18; band 1 A), placing the particles in the same density range as reported by others (Buck et al., 2004). The new construction with BiFC showed a similar migration pattern with a grey band forming about ¼^th^ the length of the density gradient from the bottom of the tube (Figure 18; band 2 A). P16sheLL particles traveled an average of 33.63 mm into the gradient, and BiFC traveled an average of 33.12 mm into the gradient, very close to the 33%/39% interface. The average migration path showed no significant differences when the centrifugation time exceeded 6 h, which was the standard procedure adopted (Buck et al., 2005).

The first few ultracentrifuged gradients were collected in ∼1 mL steps, and SDS-PAGE was briefly used to analyze all fractions with Coomassie stain to detect off-target size or other factors that could cause differential particle migration. The fractions were collected by puncturing the bottom of the tube with a thin needle (25G) about 1 fraction length below the visible grey band and allowing 1 mL to flow into the appropriately labeled tube at a time. In this sequence, the 2^nd^ fraction collected contained the majority of the viruses and the entire visible grey band (Figure 19). The middle set of bands located at the interface of the sedimentation path (Figure 18; 2A and 2B) was confirmed to be other cellular proteins expected to remain in the upper half of the tube, above the OptiPrep gradient (Figure 20; Fractions 8 and 9).

**FIGURE 19:**
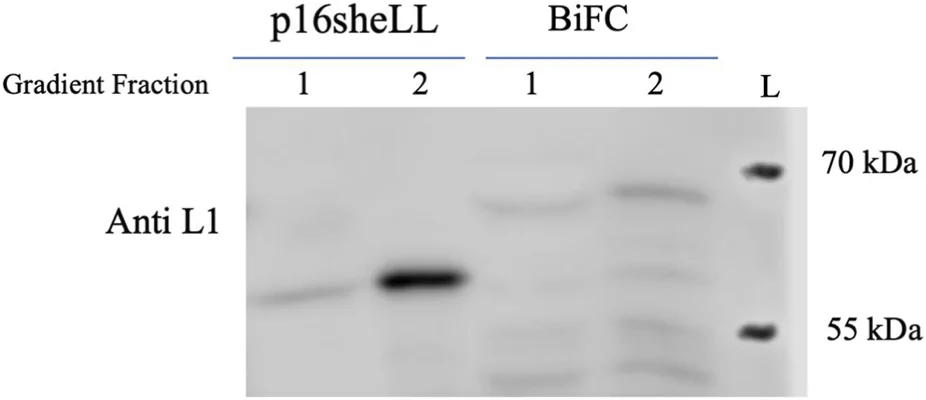
Immunoblot of collected OptiPrep fractions with peak L1 content. P16sheLL VLPs, reduced with SDS, showed L1 antibody signal at the expected size of ∼56 kDa. BiFC-VLPs reduced with SDS showed a less intense L1 band at the expected size of ∼68 kDa. Lanes 1 and 2 represent fractions 1 and 2 collected from p16sheLL-producing cell lysates after ultracentrifugation. Lanes 3 and 4 represent fractions 1 and 2 collected from BiFC-producing cell lysates after ultracentrifugation.

**FIGURE 20:**
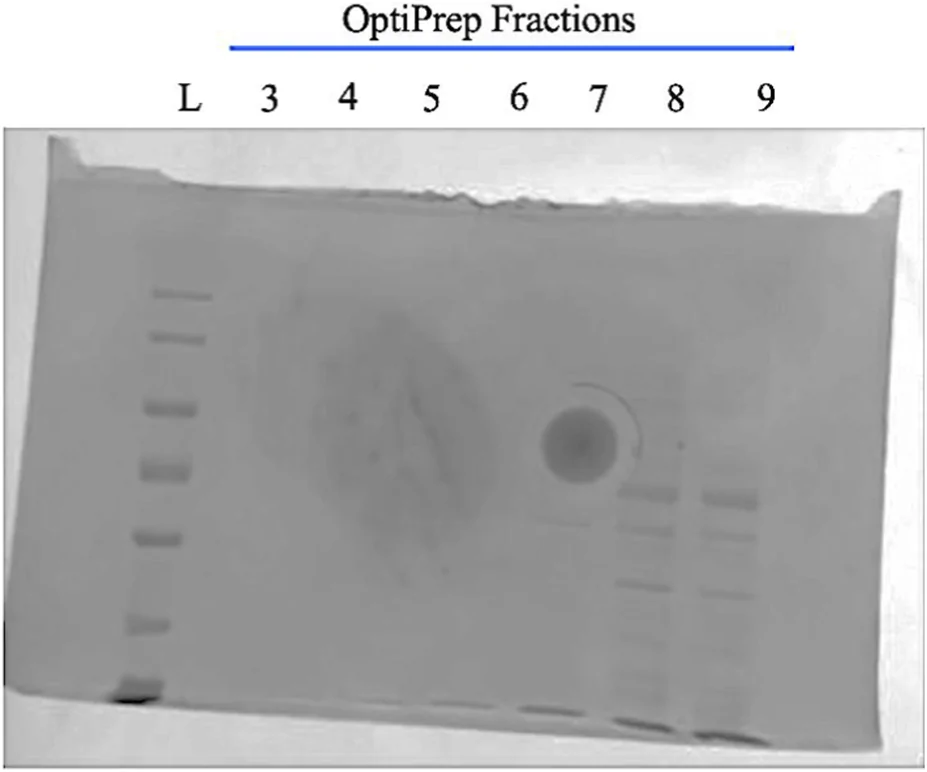
Coomassie stain of the first gradient analyzed (fractions 3–9). The first lane after the ladder is the 3^rd^ fraction collected, or the fraction directly above the fraction with a visible grey band. Fractions 7, 8, and 9 in the last three lanes contained other cellular proteins or components that did not migrate through the gradient.

However, the pattern became more consistent: L1 content in BiFC-VLP samples was spread out across the first 3 of the 1 mL fractions collected (Figure 21). A two-way ANOVA with Bonferroni correction for multiple comparisons was conducted, and significance between intensities was found between each fraction within the same treatment group (F = 75.5, DF = 2). P16sheLL-VLPs showed more significant differences and tracks with the accumulation in fraction 2 by these VLPs (p < 0.0001). BiFC-VLPs showed less significant differences between the fractions as they were more dispersed (p < 0.01 and p < 0.001). There was no significant difference in intensity across fraction 2 samples, suggesting the overall particle yield/protein (L1) concentration in fraction 2 is similar in both constructs.

**FIGURE 21:**
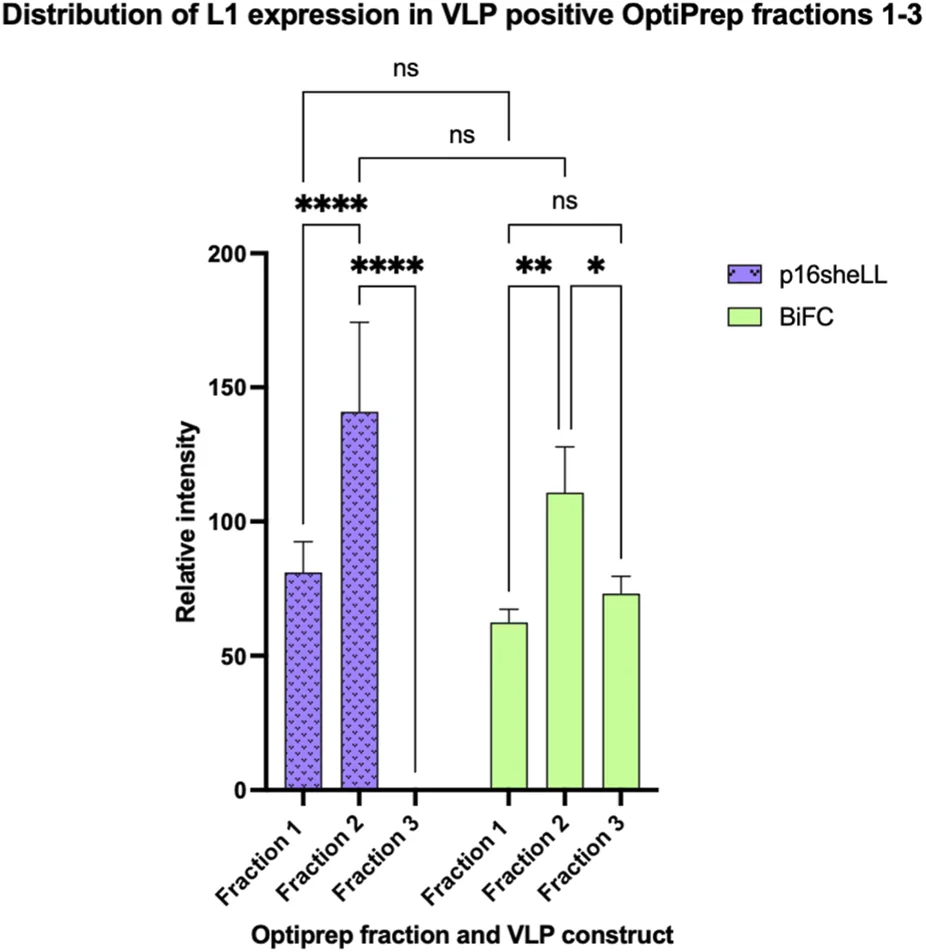
Averaged relative expression of L1 through the first three OptiPrep fractions. The mean grey value (intensity) across 3 technical replicates and 3 biological replicates for constructs p16sheLL and BiFC is plotted with error bars. A 2-way ANOVA was performed across each fraction per construct. Significance is marked by * for p < 0.05, ** for p < 0.005 and **** for p < 0.00005, and ns for p > 0.05, meaning not significant. Bonferroni correction occurred for each P value. Relative intensity was measured with Fiji. The mean grey value was normalized against background and loading control values.

The distribution of BiFC-VLPs reduced the virus concentration per mL slightly, but not enough to fall below the working concentration for an appropriate Multiplicity of Infection (MOI). Each fraction with significant L1 was imaged by TEM, and a physical count per area on the TEM grid facilitated estimation of MOI. The wide distribution of L1 expression across the OptiPrep fractions may be due to significant size variation resulting from different fluorophore fits during capsid assembly. However, a more plausible explanation is that appropriate spacing between particles and a decreased tendency to aggregate are promoted by the addition of the fluorophore with a thoughtfully designed linker. The slight increase in size from the addition of linker and fluorophore may also increase surface area, causing some larger particles to migrate higher in the gradient (Fraction 3).

### 3.6 Transmission electron microscopy analysis of BiFC-fused HPV16 VLPs

Many other groups have used TEM to characterize nanoparticles (Gaur et al., 2023). Additionally, negative stain with uranyl acetate is documented in the detection of native HPV and non-native HPV virus-like particles (Cardone et al., 2014; Buck et al., 2005). VLPs were imaged by TEM and showed distinctive HPV pentamer formation and an icosahedral shape (Figure 22).

**FIGURE 22:**
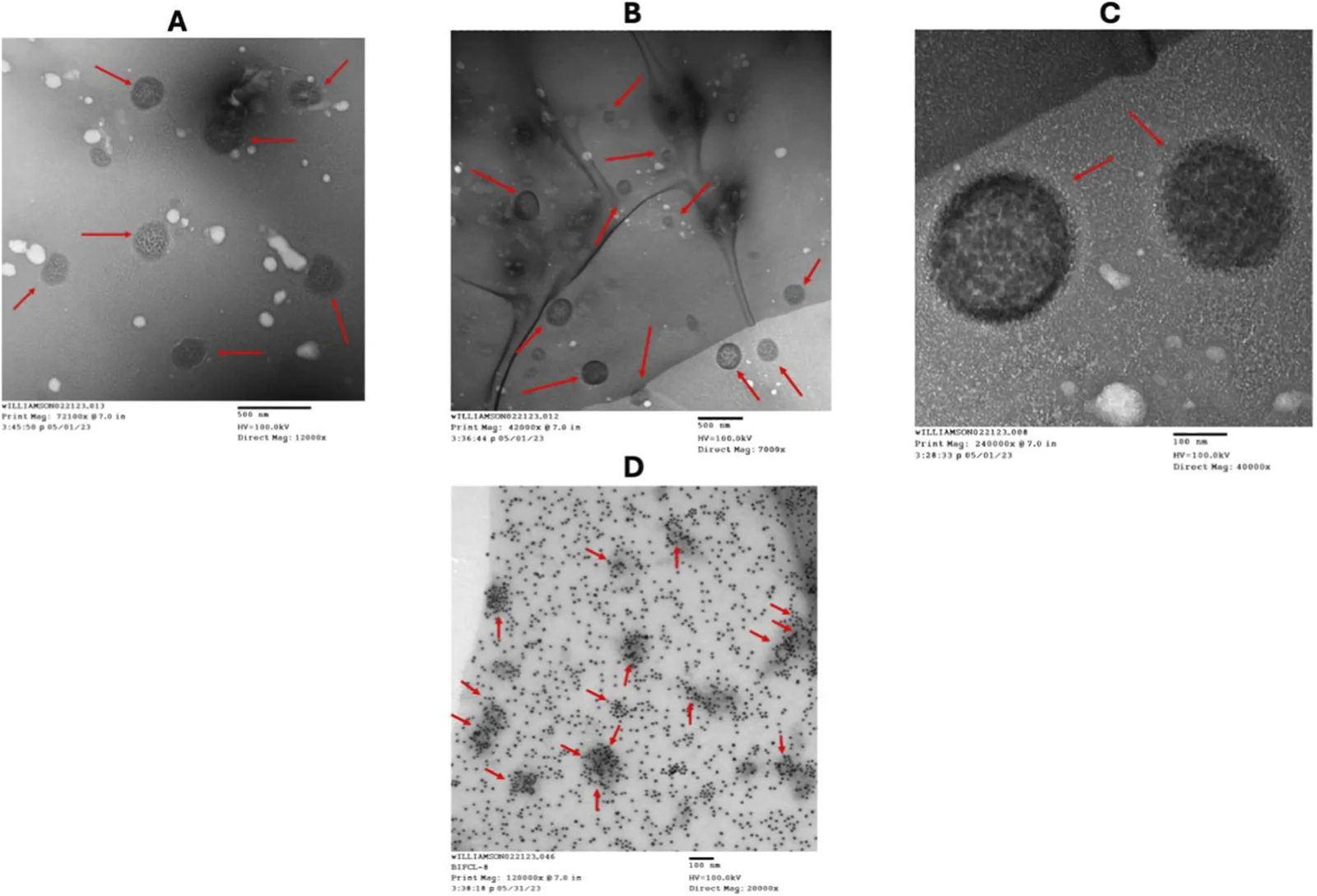
Electron Micrographs of BiFC-VLPs. **(A)** The VLPs are the darkly stained, hollow Bucky Ball-like structures (arrows). Brighter dots in the field are likely sucrose and PBS precipitate from VLP storage in OptiPrep—magnification 120000x. **(B)** Assembled L1-L2 VLPs are darkly stained. Magnification 7000x. **(C)** BiFC-VLPs at magnification 40000x. **(D)** Electron Micrograph of BiFC-VLPs at magnification 20000x. In **(D)**, grids were additionally stained with L1 antibody-conjugated 10 nm gold beads, depicted as darkly stained uniform circles on the majority of the grid (red arrows). VLPs are shown as darker shadows, about 50 nm in size, resulting from the dense, specific accumulation of gold-conjugated particles against L1. Total magnification is included as “Print Mag” under each image.

**FIGURE 23:**
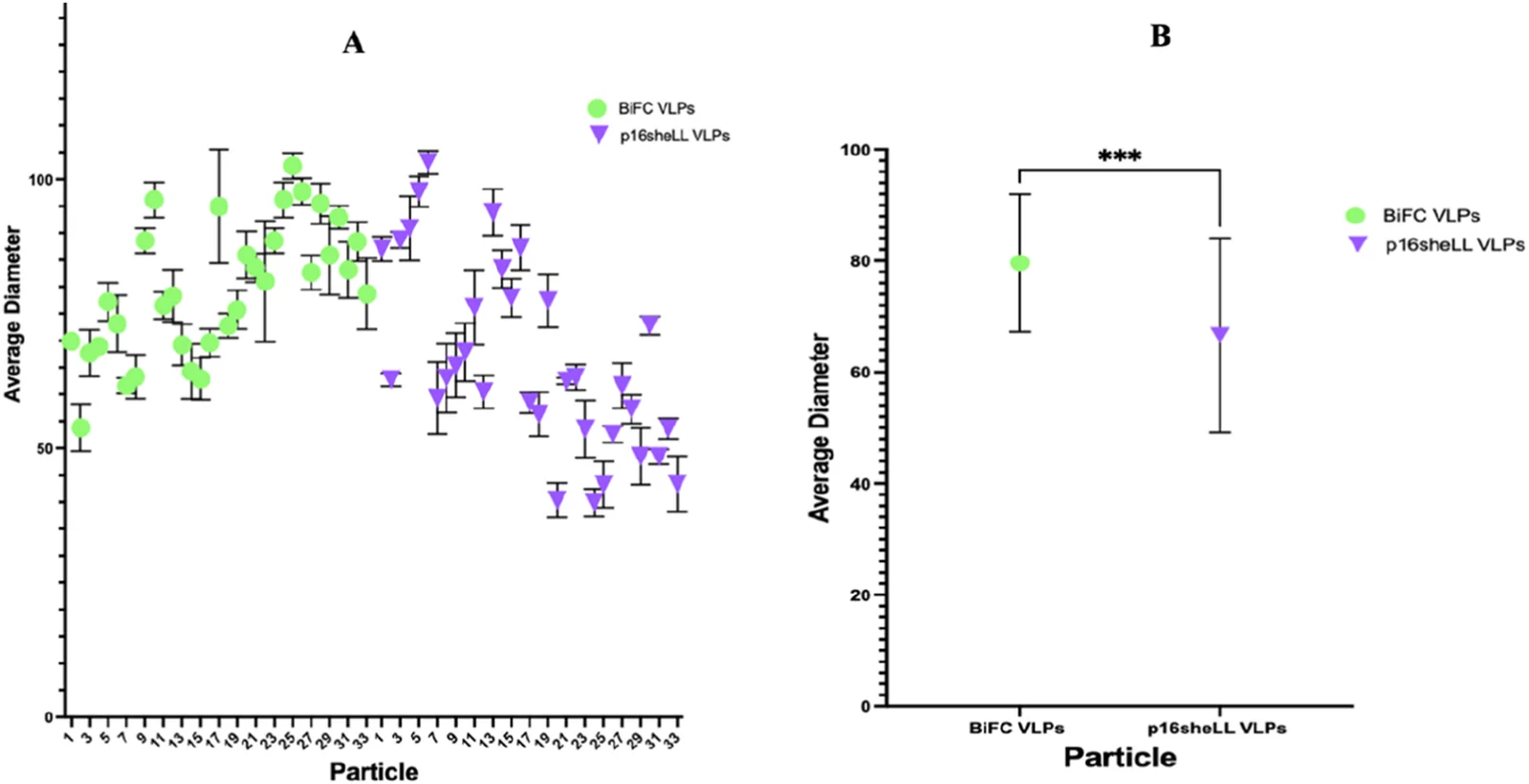
Relative diameter of each singular particle across samples and image fields. **(A)** Average diameter of individual particles with standard error across three directions of measurement. **(B)** Average diameter and standard deviation across all BiFC VLP particles (green circles) vs. average diameter of p16sheLL VLP particles (purple triangles) with T-test and Welch’s multiple comparison correction used to determine significant difference between p16sheLL and BiFC VLP size distributions. *** = p < 0.0005.

The scale bar for each image and each particle with distinct morphology was measured with the line selection tool in Fiji. Each measurement in pixels was transformed to approximate length in nanometers by proportional comparison with scale bar measurements. To determine uniformity of shape, the diameter was measured by drawing a line horizontally (X), vertically (Y), and diagonally (Z) through the particle’s central point for each. Mean grey intensity peaks along the length of the line selection, allowing appropriate pixel length estimation from the intensity difference between the valleys. Diameter of singular particles that were not aggregated nor attached to debris, from multiple imaging fields and biological replicates, is shown in Figure 23. The diameter in each direction was averaged per particle, and the standard error between directions of measurement within a single particle is represented by error bars. Welch corrected t = 2.927 with DF = 55.57. Variance in diameter within a single particle can be from processing or 2D image flattening, making the particles more oval than spherical. This method of measuring 2D size using ImageJ is used in other methods and compared with 3D measurements from light-scattering instruments (Ardon-Dryer et al., 2015).

### 3.7 Determining VLP size with Dynamic Light Scattering

The average diameter and intensity are displayed for each “good quality” (0.01< PDI <0.5) run (Danaei et al., 2018) (Figures 24–26).

**FIGURE 24:**
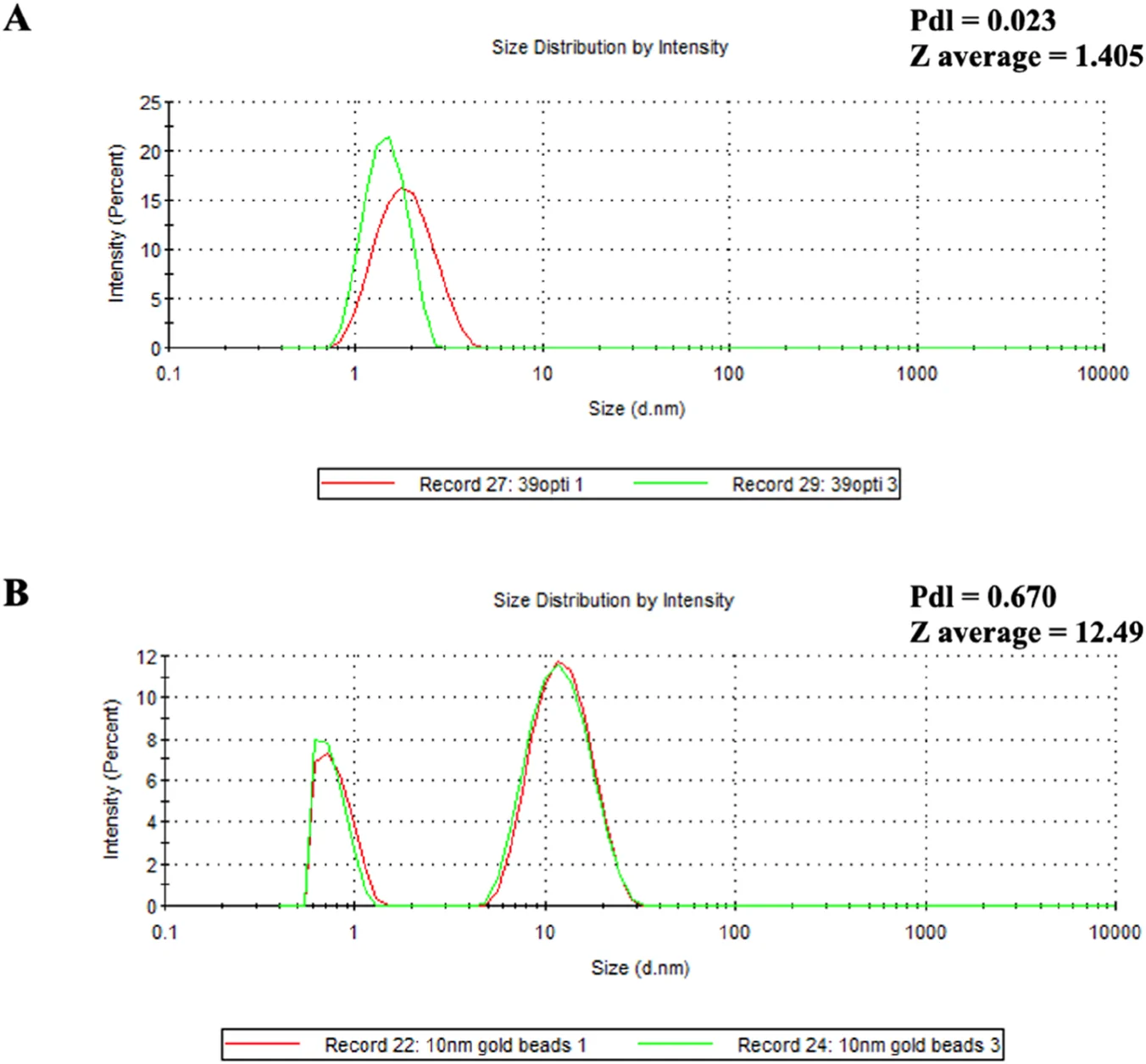
Average diameter distributed by intensity of measured particles in technical control samples. **(A)** OptiPrep at 39% in 0.8 M DPBS, estimated at ∼1 nm, was used as a background control for relative sample purity. **(B)** 10 nm gold beads, which served as the technical control for particle-size detection and were measured at approximately 10 nm. The area under each curve represents the relative intensity distribution of particles by size. PDI values are shown for each control sample.

**FIGURE 25:**
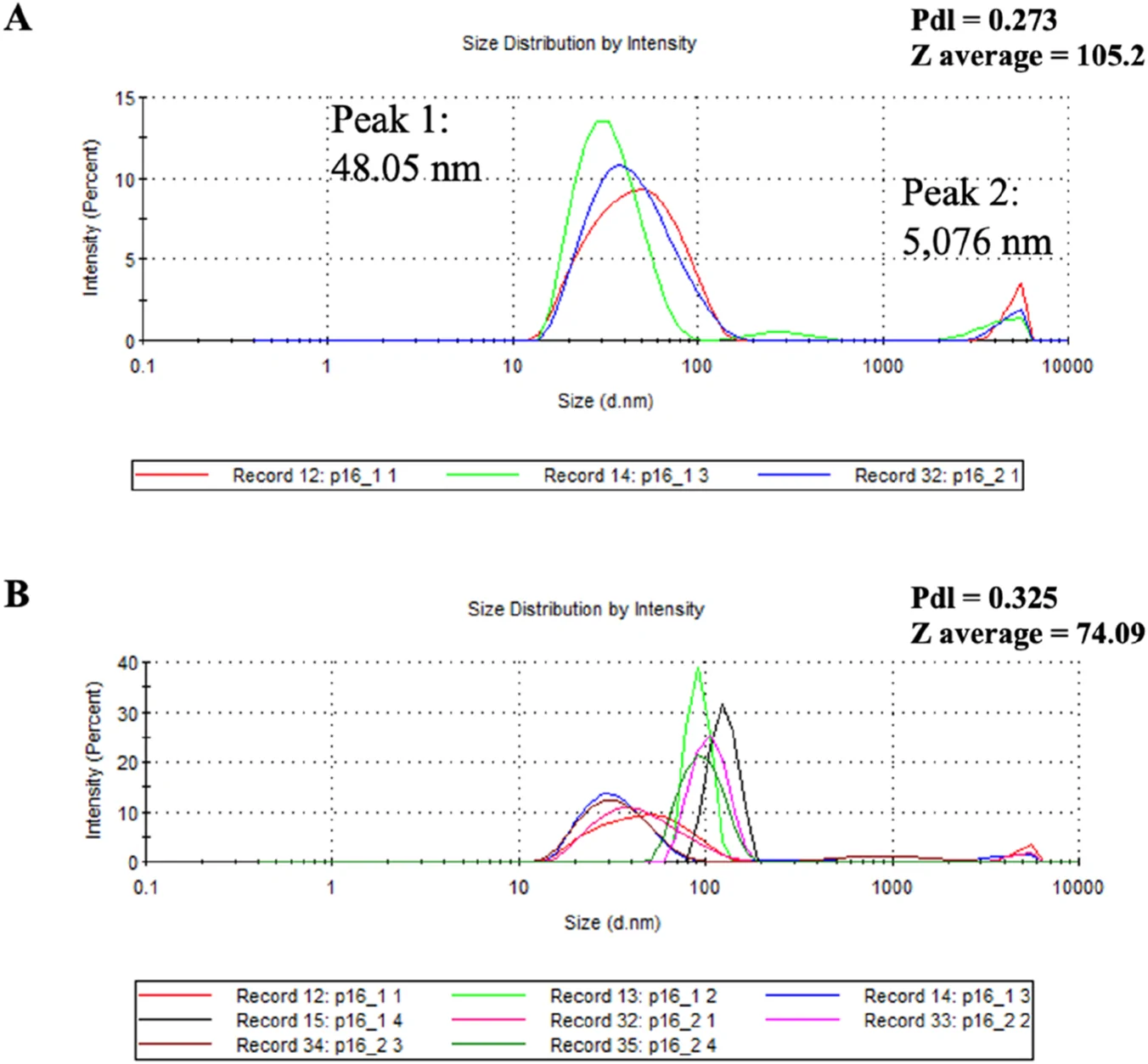
Average diameter distribution by particle intensity in Optiprep^TM^ Solution for positive control p16sheLL, which produced HPV VLPs. **(A)** Size measured with a forward scatter detector. **(B)** Size measured with both forward and backward scatter detectors.

**FIGURE 26:**
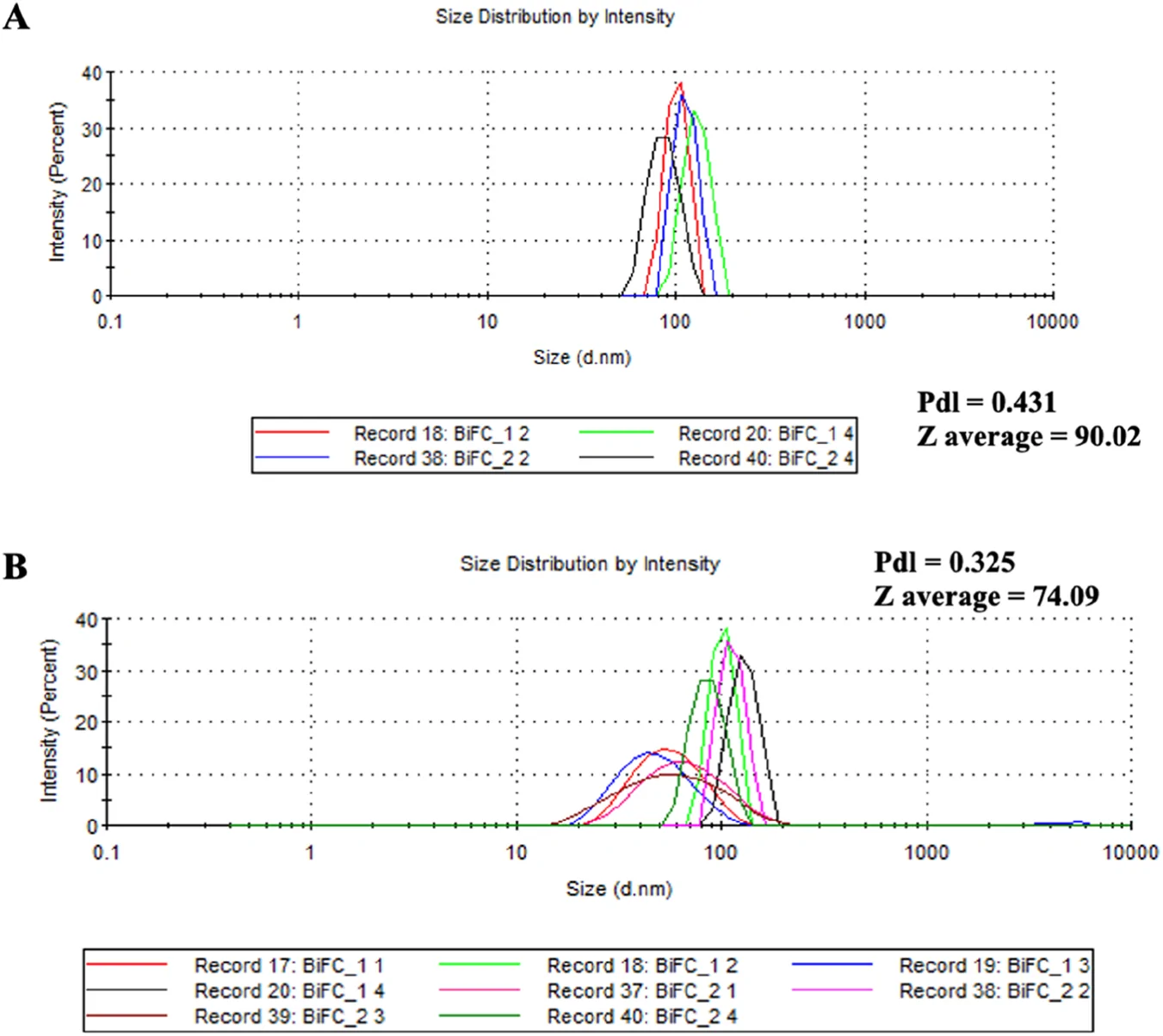
Average diameter distribution by particle intensity in Optiprep^TM^ Solution for the positive controls pBiFC-L1-VC155 and pBiFC-L2-NV173, which produced HPV VLPs. **(A)** BiFC sample runs of good quality are measured through the forward scatter detector. **(B)** All BiFC sample runs measured by both forward and back scatter detectors.

Both forward and back scatter angles were used to get the maximum amount of information possible. Small particles (<15 nm) with uniform shape and a decent concentration will exhibit isotropic (equal amounts of light scatter in all directions) detection at both angles; the 10 nm gold beads would represent such particles. Uniform detection was not recorded with the 10 nm beads, but the size calculation between forward and backscatter detection was the most similar within replicates of that sample type. Comparing the two angles can help distinguish heterogeneous particles in a solution (Austin et al., 2020), as larger particles or aggregates would scatter more light in the forward direction (12.8°). Backscatter detection at 173° can help distinguish contaminant particles and reduce the effects from multiple scattering of an already scattered light path (Malvern Instrument User Guide). In Table 2, the average diameter and PDI were accumulated from each peak intensity and mean diameter per kilocounts per second (kcps not displayed). The average diameter was calculated for each technical replicate, consisting of 9 sequential 10-s reads (Table 2, Column 5). The average diameter between 3 biological replicates was calculated for each angle of measurement (Figure 27).

**TABLE 2:** Raw data from Zetasizer ZSP displaying each peak detected.

Record	Type	Sample name	T ( ∞ C)	Ave (d.n	PdI	Pk 1 mean int (d.nm)	Pk 2 mean int (d.nm)	Pk 3 mean int (d.nm)	Pk 1 area int (percent)	Pk 2 area int (percent)	Pk 3 area int (percent)	Aggregation index	Scattering angle ( ∞ )
1	Size group	BSA 1 mg/mL	​	​	​	​	​	​	​	​	​	6.92	173
2	Size	BSA 1 mg/mL 1	25	1.861	0.322	1.72	56.67	0	82.2	17.8	0	​	173
3	Size	BSA 1 mg/mL 2	25	18.94	1	139.6	1.506	0	87.1	12.9	0	​	12.8
4	Size	BSA 1 mg/mL 3	25	1.945	0.342	1.739	56.91	0	80.1	19.9	0	​	173
5	Size	BSA 1 mg/mL 4	25	11.23	1	134	1.636	0	81.9	18.1	0	​	12.8
6	Size group	BSA DTT 1 mg/mL	​	​	​	​	​	​	​	​	​	2.26	173
7	Size	BSA DTT 1 mg/mL 1	25	2.449	0.42	1.993	48.29	4815	76.2	20.1	3.7	​	173
8	Size	BSA DTT 1 mg/mL 2	25	7.502	I	58.01	0.8897	0	84.3	15.7	0	​	12.8
9	Size	BSA DTT 1 mg/mL 3	25	2.539	0.452	1.909	60.06	3348	70.4	25.1	4.5	​	173
10	Size	BSA DTT 1 mg/mL 4	25	8.782	1	107.5	1.507	0	79.7	20.3	0	​	12.8
21	Size group	10 nm gold beads	​	​	​	​	​	​	​	​	​	14.6	173
22	Size	10 nm gold beads 1	25	3.025	0.674	12.81	0.7945	0	73.1	26.9	0	​	173
23	Size	10 nm gold beads 2	25	25.43	1	117.2	6.328	0	91.9	8.1	0	​	12.8
24	Size	10 nm gold beads 3	25	2.995	0.67	12.49	0.7558	0	74.7	25.3	0	​	173
25	Size	10 nm gold beads 4	25	68.5	0.614	114	0	0	100	0	0	​	12.8
26	Size group	39opti	​	​	​	​	​	​	​	​	​	4.6	173
27	Size	39opti 1	25	1.718	0.1	1.912	0	0	100	0	0	​	173
28	Size	39opti 2	25	8.126	I	69.39	1.121	0	84.4	15.6	0	​	12.8
29	Size	39opti 3	25	1.405	0.023	1.476	0	0	100	0	0	​	173
30	Size	39opti 4	25	9.381	1	213.8	1.116	0	80.9	19.1	0	​	12.8
11	Size group	p16_1	​	​	​	​	​	​	​	​	​	3.95	173
12	Size	p16_11	25	46.71	0.358	50.36	5076	0	93.2	6.8	0	​	173
13	Size	p16_12	25	222.6	1	92.04	0	0	100	0	0	​	12.8
14	Size	p16_13	25	28.36	0.449	34.5	4255	289.6	91	5.9	3.1	​	173
15	Size	p16_14	25	148.9	1	125.8	0	0	100	0	0	​	12.8
31	Size group	p16_2	​	​	​	​	​	​	​	​	​	1.83	173
32	Size	p16_21	25	35.56	0.473	48.05	4782	0	94.9	5.1	0	​	173
33	Size	p16_22	25	105.2	0.273	106.6	0	0	100	0	0	​	12.8
34	Size	p16_23	25	27.7	0.452	34.85	1075	0	89	11	0	​	173
35	Size	p16_24	25	74.09	0.325	97.52	0	0	100	0	0	​	12.8
16	Size group	BiFC_1	​	​	​	​	​	​	​	​	​	2.77	173
17	Size	BiFC_11	25	44.49	0.271	57.64	0	0	100	0	0	​	173
18	Size	BiFC_12	25	167.1	1	101.2	0	0	100	0	0	​	12.8
19	Size	BiFC_13	25	40.64	0.263	49.9	4571	0	98.1	1.9	0	​	173
20	Size	BiFC_14	25	153.9	I	129.3	0	0	100	0	0	​	12.8
36	Size group	BiFC_2	​	​	​	​	​	​	​	​	​	1.42	173
37	Size	BiFC_21	25	51.57	0.288	71.28	0	0	100	0	0	​	173
38	Size	BiFC_22	25	141.9	I	112.8	0	0	100	0	0	​	12.8
39	Size	BiFC_23	25	44.15	0.315	64.27	0	0	100	0	0	​	173
40	Size	BiFC_24	25	90.02	0.431	86.52	0	0	100	0	0	​	12.8

**FIGURE 27:**
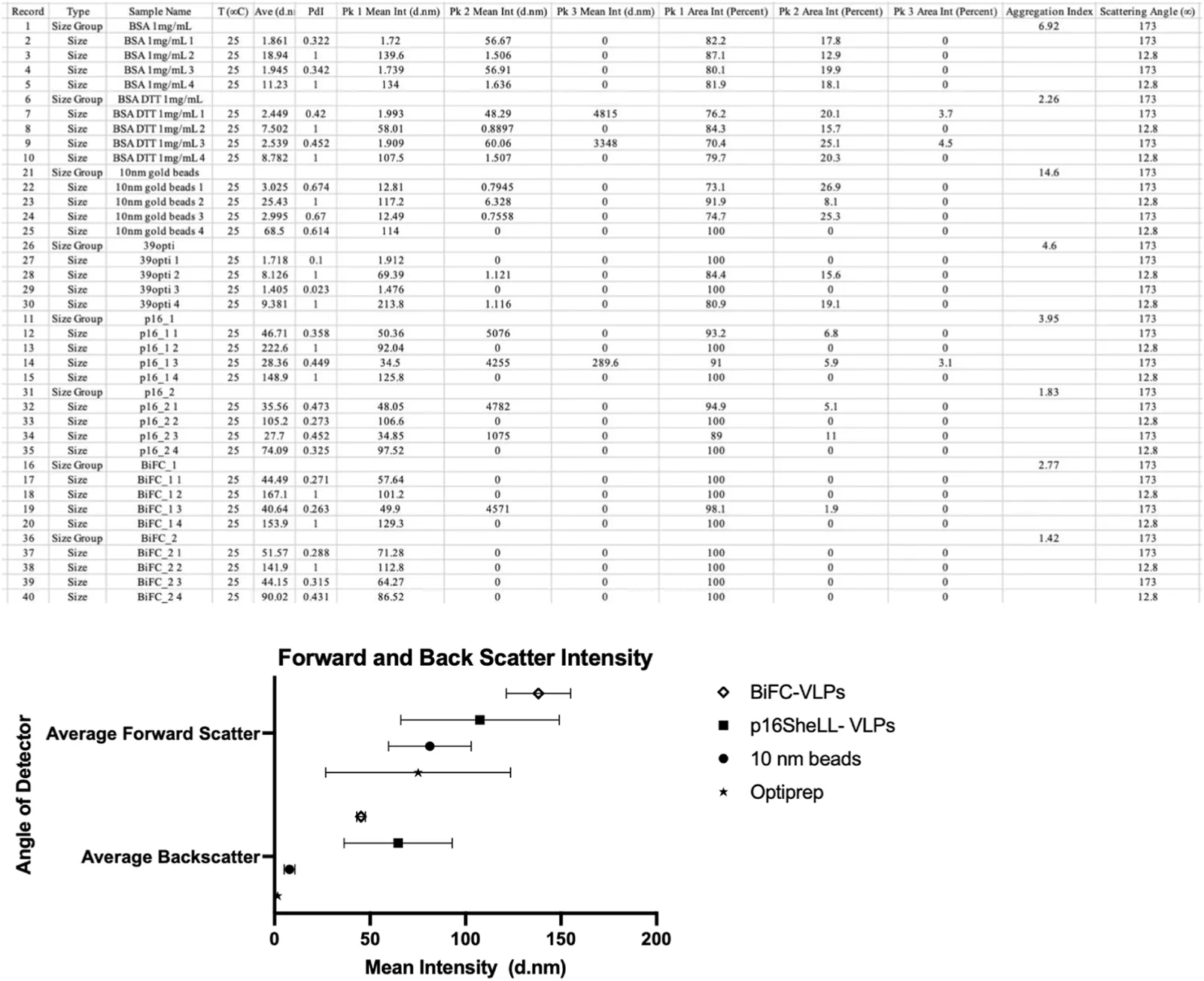
Mean diameter from forward and back scatter intensities. Average size calculated by light scatter detected in the forward or backscatter positions for each sample type. The X-axis shows the mean diameter in nanometers, calculated using the Stokes-Einstein equation with the Zetasizer. Sample types include newly constructed BiFC capsids, positive control p16sheLL capsids, and technical controls: 10 nm beads in Optiprep™. Mean and standard error were calculated across technical and biological replicates.

Because each sample type is expected to differ in size, only the within-group standard error was measured. Backscatter intensity for gold beads and OptiPrep solution matched the expected values of these controls with little variance. OptiPrep was shown to be highly monodisperse, with a PDI of 0.02, whereas a PDI <0.05 is rarely observed in lower-purity samples. From this, we are confident that there is no interference from the storage medium in DLS measurement techniques. Backscatter and forward scatter of BiFC-VLPs in OptiPrep solution showed less size variance than p16sheLL. BiFC and p16sheLL VLPs measured with backscatter were placed near the 50 nm mark, matching the results of 2D TEM image analysis. More in-depth comparisons between all particle observations are discussed in Chapter 4.

P16sheLL particles in OptiPrep showed the greatest peak variation, with averages of 50, 100, and 4000 nm. BiFC particles in OptiPrep showed the strongest intensity, and averages were uniform between 50 and 100 nm. These results track with the previously reported results in Section 3.6.

### 3.8 Analysis of potential encapsidated DNA

Potential DNA content in the VLPs was analyzed using a DNA extraction kit (Qiagen PCR cleanup kit) and a disassembly buffer containing 10 mM DTT. Both preparations showed DNA levels below the limit of detection prior to PCR amplification. PCR amplification of potential contaminants showed no distinct DNA bands on agarose gel analysis (Figure 28). PCR primers for E6 and SV40 large T sequences were designed with slightly reduced specificity for template binding, and a 2 °C lower annealing temperature was used to increase the likelihood of partial sequence recovery. Only the ladder and loading dye front and primers appeared on the gels from capsids immediately after isolation from the cell host, as expected.

**FIGURE 28:**
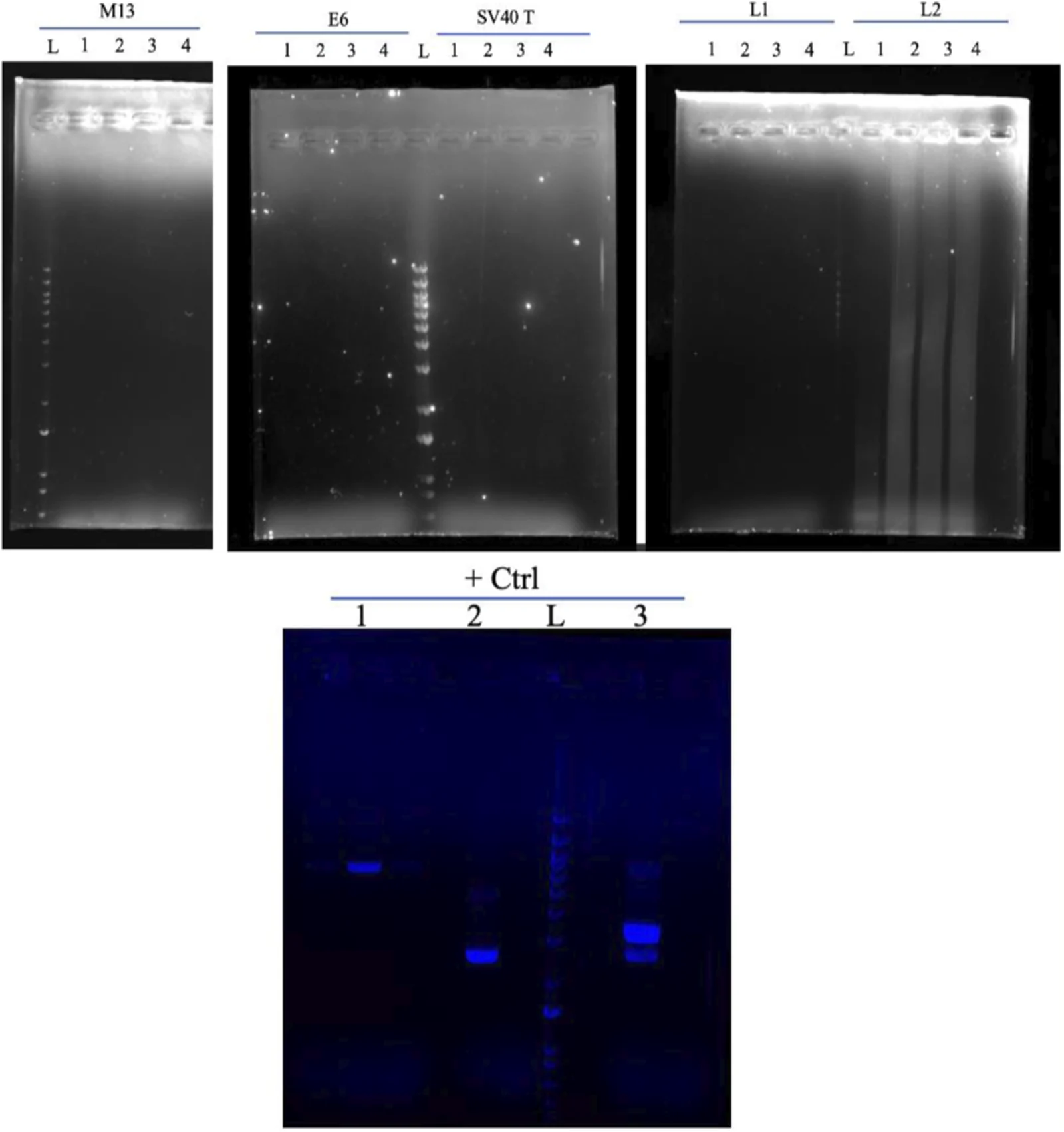
Random and specific PCR analysis of encapsidated DNA. DNA was analyzed with 1% agarose gel electrophoresis. A lack of distinct bands in the experimental group (top row) gels demonstrates the safety of the capsids with no detection of any significant HPV or SV40 genome portions, no significant self-packaging of BiFC plasmids, and overall undetectable amounts of DNA, suggesting these capsids are likely empty. Positive control samples are displayed (Bottom row). Lane 1 shows nonspecific binding of the M13 primer pair to the p16sheLL plasmid. Lane 2 shows amplification of the E6/E7 in-house plasmid mix containing portions of the ∼2.5 kb E6/E7 ORFs. Lane 3 is amplification of SV40 large T antigen from an in-house mix of full-length (∼4 kb) and truncated genes isolated from polyclonal bacterial stocks. L1+VC155 and L2+VN173 positive control not shown, that primer pair is validated in the supplementary.

The bottom row in Figures 3–29 shows a positive control using p16sheLL collected capsids exposed to plasmid DNA mixtures in the presence of DTT. Based on normal capsid formation imaged by TEM, it is assumed that previously reported disassembly buffers used by other groups (Cerqueira et al., 2016) can be used to encapsidate plasmid DNA in a cell-free system. Reassembly of the capsid proteins, with or without added plasmid, was performed by changing the buffer conditions to a reducing environment before the positive control samples were processed, as in the experimental groups, for DNA isolation. This experiment did not confirm encapsidation of these plasmid mixes into the positive-control p16sheLL VLPs, which is a limitation. Regardless, the positive control samples contained both VLPs and coding sequences that were successfully amplified by the primer pairs described in ST1.

**FIGURE 29:**
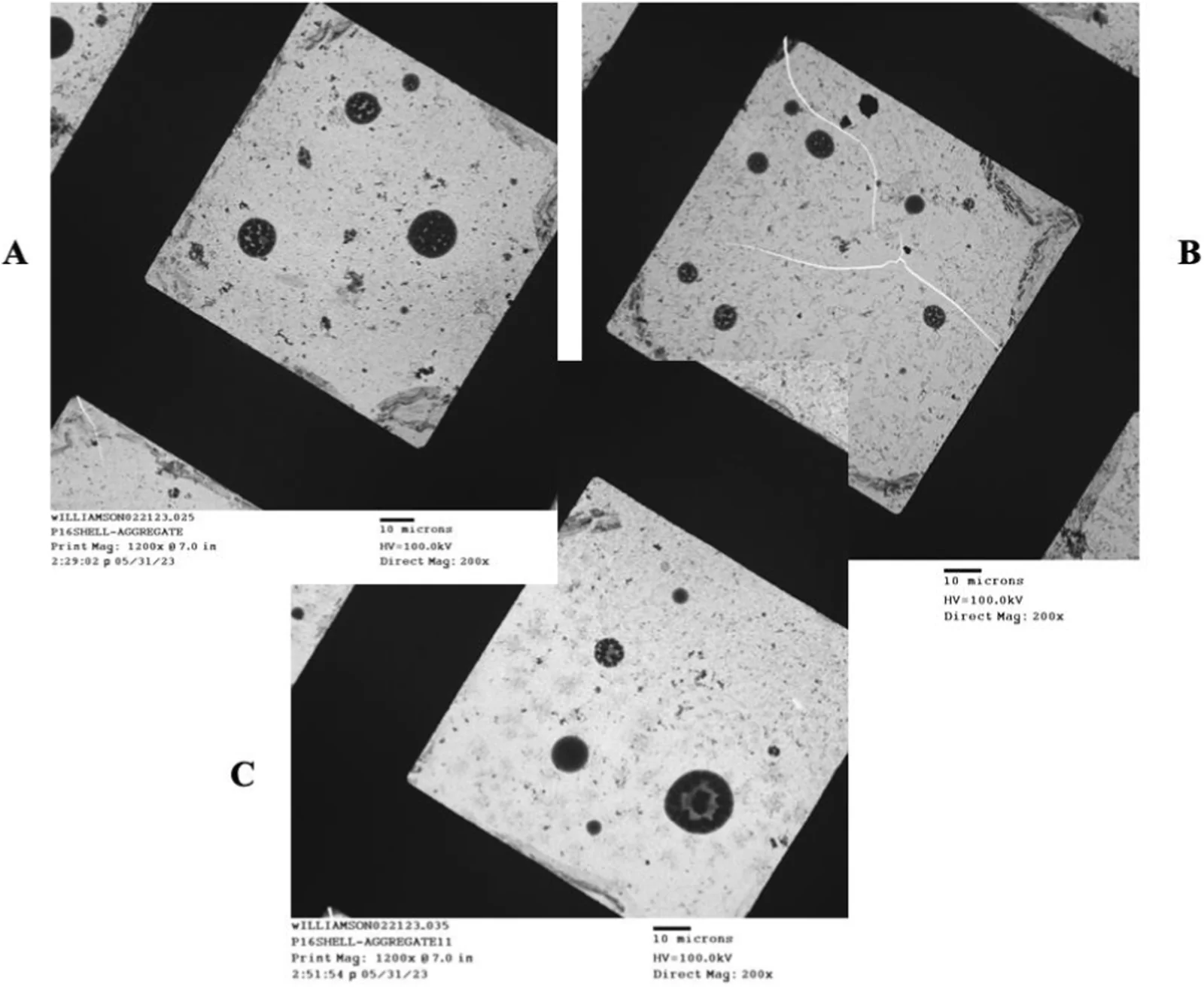
Electron micrographs of p16sheLL VLPs in unwashed TEM samples. **(A–C)** Display different boxes within the same grid. Images taken at a minimum ×200 magnification to demonstrate size.

## 4 Discussion

A large part of creating a new nanoparticle is developing methods for both constructing and characterizing said nanoparticle. This is especially important when testing the particles for applications to Human Health. One problem with recent advances in nanotechnology and viral vector technology is the lack of live-cell and real-time nano-tracking of particles during *in vitro* production and investigations.

### 4.1 Discussion of considerations for new capsid construction and validation

#### 4.1.1 Producer cell choice and accessibility of the platform

HPV16-BiFC VLPs were harvested and purified as described by other research groups (Buck et al., 2006; Buck et al., 2004; Chen et al., 2000; Kim et al., 2016; Li et al., 2022; Schellenbacher et al., 2017; Slupetzky et al., 2007; Zhao et al., 2022). In this study, 293T cells were selected as the host cell model for plasmid transfection and intracellular viral capsid formation, as they are widely used as producers for a range of VLP systems (Han et al., 2025; Hołubowicz et al., 2025). Unlike current methods that rely on 293 TT cells (Cerqueira et al., 2017), the modified coding regions used in this project were placed under a general CMV promoter. Because CMV is a broadly active mammalian promoter, its use may improve construct compatibility across commonly available producer cell lines and reduce the need for specialized SV40 large T-expressing systems. Additionally, this design has the potential to increase accessibility of HPV16 VLP production by reducing dependence on specialized producer systems. Following production, isolated VLPs were analyzed for the presence of the Large T coding region as a quality control step, as unintended packaging of producer cell-associated sequences would be undesirable in downstream applications (De et al., 2023). Although HeLa cells represent another easily transfected producer cell type, there is a substantial risk of packaging E6/E7 genomic material associated with that cell line. Because of this, HeLa cells are not ideal for producing empty VLPs intended for therapeutic use. However, HeLa cells may still be useful in experimental settings where larger portions of the HPV genome are intentionally packaged for infectious studies.

#### 4.1.2 Evidence that BiFC modification does not disrupt capsid maturation

Successful, real-time capsid assembly was characterized by cell swelling and a peak in BiFC signal intensity in 293T cells after ∼56 h (Figure 15). In this figure, clearly swollen cells are overlaid with BiFC signal, supporting the interpretation that productive capsid formation occurred during this time window. Previous studies reported that 293 TT cells, which contain a stable extra copy of large T antigen and express p16sheLL at high copy number, “swell up like beach balls” (Buck et al., 2004). In the present study, flow data from standard 293T cells showed less size variation for the positive control p16sheLL vector, and immunoblotting confirmed a slightly lower protein yield, likely reflecting lower large T expression in the regular 293T background. By 96 h, the flow data showed substantial cellular debris, suggesting that cells loaded with capsids had begun to burst following transient transfection with both constructs.

Capsid maturation was further supported by the use of an alkaline lysis buffer optimized with ammonium sulfate, which allowed maturation when lysis occurred for 24–36 h at 37 °C (Kopitak et al., 2025). Formation of the HPV icosahedral structure depends on disulfide bond formation between L1 subunits, a process that occurs relatively slowly (Buck et al., 2013). Because there were no significant changes in the overall charge of L1 pentamers or the total capsid, the pH of all buffers used throughout lysis, storage, and dilution was maintained. The icosahedral pattern is initiated by disulfide bonding between residues Cys175 and Cys428 (Wang et al., 2020). L1 trimers are often seen in reducing Western blot analysis without appropriate enzymatic digestion or reduction of samples containing assembled viral particles or partially assembled polypeptide fragments (Trejo-Cerro et al., 2026). In this study, the consistent appearance of L1 dimers and trimers in L1 immunoblots (Figure 19) suggested that BiFC-constructed capsids retained disulfide bond behavior similar to that expected of normally assembling particles. Together, these findings support the conclusion that BiFC modification did not disrupt the normal capsid properties required for proper assembly and maturation.

It remains unclear whether L1-VC155 and L2-VN173 molecules produced in separate cells can assemble during lysis and maturation. Native HPV capsid assembly occurs intracellularly, although cell-free assembly of papillomavirus-like particles has also been reported (Cerqueira et al., 2016). Thus, proper maturation and lysis could increase the efficiency of these plasmids in producing fully formed particles. Excess L2 expression compared to native and p16sheLL, which involves L1 and L2 at near-equimolar concentrations, could facilitate maximum L2 saturation per particle and, concurrently, maximum fluorophore intensity per particle, but this potential remains unexplored and is beyond the detection methods provided in this study.

#### 4.1.3 Heterogeneity of the transfected population and implications for particle composition

Each cell in the transfected population falls into one of four categories, yielding a range of expression levels. This includes 293T cells with no plasmid uptake, pBiFC-L1-VC155 only, pBiFC-L2-VN173 only, or both pBiFC-L1-VC155 and pBiFC-L2-VN173. Immunoblotting of total cell lysate was used to quantify total protein; however, this does not directly inform the estimation of fully formed capsid yield. The use of fluorescent imaging (Flow and ICC) enabled more quantification of the BiFC signal over time. ICC also showed overlap between BiFC signal and L1 antibody staining relative to L1 antibody staining alone, allowing estimation of the ratio of L1/L2-containing versus L1-only capsids produced in 293T cells. This ratio provides a more informative estimate of transfection efficiency, particularly for the subset of cells that successfully received both plasmids. Because L1 particles can assemble into pentamers and support loose capsid formation with or without L2 (Chen et al., 2000), there is substantial potential for a high proportion of L1-only VLPs relative to L1/L2 VLPs, both across the overall transfected population and within individual cells expressing both plasmids. Mixed particle populations of this type remain less well characterized. However, there is currently no evidence to suggest that a heterogeneous mixture of VLP types is inherently less effective for packaging or targeting, or that it introduces additional safety concerns. Practical separation of L1-only and L1/L2-containing particles remains challenging, as the structural difference between these capsid types is limited to only a slight reduction in diameter associated with increased capsid tightness. Because L2 is the minor capsid protein, variation in L2 incorporation also does not substantially alter total particle weight.

#### 4.1.4 Imaging and flow-based detection of BiFC capsid formation

Overall, the ratio of L1-only to L1/L2 capsids became more consistent at higher magnification and with more specific excitation/emission filters. The highest fluorescence detection was recorded with the Attune flow cytometer, which is well-suited for high-throughput flow virometry applications. The wavelengths of the BL1 filter, together with the separation of cells as they pass through the laser in the optical cell, increased the recorded transfection efficiency and apparent L1/L2 capsid production. The Attune measured approximately 18% of the total population as L1/L2 capsid-producing cells, whereas the Nikon, Olympus, and Leica scopes averaged approximately 11.1%, 7.9%, and 3.8%, respectively. This difference is not unexpected, as flow cytometry measures the entire population, whereas microscopy requires averaging across multiple fields of view.

A specific YFP filter, such as the one used on the Attune and Nikon systems, provided the most accurate approximation of VLP formation efficiency, which was expected. The Olympus scope with a FITC filter was the next-best option. When a YFP filter is unavailable, the BiFC signal can still be detected within the FITC range, and accuracy improves with increased magnification. The GFP filter in the Leica had the least spectral overlap with YFP, so the number of BiFC-positive cells with sufficient intensity above the background is lower.

In this specific comparison, the phase-contrast and brightfield images captured by the Leica were substantially better for cell border visualization and improved the accuracy of cell counting per image relative to the Olympus system. In the Olympus BX-51 instruments available in the department, dust particles on the phase-contrast lenses partially obstructed the camera view, as shown in Figure 13, where multiple dark spots and lower contrast between cell borders are visible compared with Figure 14. For Alexa 633 detection, both PI and Texas red filters performed with similar accuracy. TRITC detection of Alexa 633 was also tested on the Olympus BX-51 using the same filter wheel as PI and FITC, but TRITC showed the lowest accuracy because of reduced spectral overlap, so PI was used for subsequent samples.

Importantly, BiFC VLP capsid formation remained detectable after formalin fixation and Triton X permeabilization, with sufficient preservation for L1 antibody binding in immunocytochemistry applications. Together, these findings indicate that BiFC VLP capsid formation can be imaged across multiple spectral ranges and instrument types, making this method widely accessible while remaining ideal for modern, high-throughput instruments.

### 4.2 Discussion of particle size, yield, and aggregation

#### 4.2.1 Particle size and morphology

It is important to note that 2D flattening of a 3D icosahedral particle can make it appear larger in TEM images. For this reason, diameters measured from 2D TEM images were used primarily to provide a rough assessment of particle uniformity, including sphericity and flattening, across the population. The relatively small error bars among diameter measurements taken within the same particle suggest that individual particles were fairly uniform in shape. Similar variation across diameter directions was also observed in p16sheLL particles, indicating that BiFC-VLPs retain an icosahedral morphology comparable to that of the positive control and undergo a similar degree of flattening during 2D imaging.

Among individual particles, the observed size distribution ranged from approximately 55–100 nm for BiFC-VLPs and 35–105 nm for p16sheLL VLPs. The addition of the BiFC components to the capsid proteins increased the overall particle size by approximately 1.5-fold relative to p16sheLL capsids, which averaged ∼50–55 nm. Notably, the overall size range was broader in the p16sheLL group than in the BiFC group.

#### 4.2.2 Estimation of particle yield and MOI relevance

Particle count per unit area of the grid was used to generate a rough estimate of the concentration of viruses per mL recovered following ultracentrifugation. Estimating the number of HPV VLPs produced in each sample allows comparison with theoretical yield and with other methods of particle measurement, which together can help validate characterization of these novel particles and provide an approximate total titer. As described in the previous sections, transfection efficiency was first used to estimate the number of cells containing the virus. This calculation was then extended by averaging the number of viruses observed per grid area, which was used to estimate the number of viruses present per 10 μL of the 1 mL fraction collected. The final step was to divide the estimated number of viruses per mL by the concentration of virus-containing cells per mL loaded onto the density gradient.

Scale bar proportion was also used to calculate the total area imaged on the grid. Approximately 20 viruses were counted within a 9.08 × 10^−6^ mm area, and this ratio was compared with x/3.05 mm, representing the full grid area. Based on this estimate, 67.2 × 10^5^ BiFC virus particles were calculated per 20 μL of sample loaded onto the grid, corresponding to an estimated yield of 33.59 × 10^7^ particles in a 1 mL fraction. Using these values, 2 μL of the undiluted VLP-containing fraction was added per 100,000 cells to maintain a multiplicity of infection (MOI) of ∼6.7. This falls within the suggested MOI range of 0.1–10 (Shabram and Aguilar-Cordova, 2000).

Expected particle count and individual particle mass, assuming that all expressed L1 proteins matured into capsids, were estimated as follows:

360 L1 molecules * 67,842 g/mol=2,442,337.9 g*mol/viral particles per mol

2,442,337.9 g*mol/particles/mol divided by Avogadro’s number (6.022 × 1023 molecules/mole) = 2.8 × 10–14 g/particles (1).

The total protein measured by BCA could then be multiplied by the L1 immunoblot ratio to estimate the amount of L1 in the total cell lysate. After unit conversion, the resulting L1 concentration could be divided by the final particle-per-gram estimate to approximate the expected particle number. Comparison of these expected values with particle counts obtained from TEM and light-scattering analyses may help estimate the extent to which late protein expression does not mature into complete capsids.

#### 4.2.3 Aggregation behavior and sample-state dependence

The attachment of BiFC proteins to the capsid resulted in slight differences in apparent 2D shape distortion compared with p16sheLL-produced capsids. However, a large proportion of the particles showed similar distortion patterns, suggesting that BiFC-VLP assemblies remained structurally uniform across the sample. Nearly all BiFC replicates showed an average-diameter peak at 100% intensity, indicating highly similar light scattering behavior across the population and suggesting relatively uniform particles with acceptable purity and concentration. BiFC-VLP samples were prepared in the same manner as p16sheLLVLPs, stored at −80 °C, and handled as suggested in the original protocol provided by (Buck et al., 2005). In contrast, most p16sheLL samples showed two intensity-derived size peaks and lower PDI in backscatter results. Taken together, these findings suggest that p16sheLL samples contained either larger VLP aggregates or incompletely separated VLPs associated with producer cell lysates through electrostatic interactions. Viral particle aggregation is not unusual, and complications arising from it are still being reported in research applications (Pradhan et al., 2022).

Some grids were not washed after the viral attachment step during TEM preparation. Because this particle construct was novel, its charge and interaction properties were unknown, and excessive washing could have disturbed the sample. Grids were therefore prepared both with and without washing after application of the virus supernatant. Addition of BiFC appeared to significantly reduce particle aggregation relative to both washed and unwashed p16sheLL samples (Figures 29–31). Particle adhesion and aggregation also affected downstream measurements. This was reflected in the Zetasizer data, where p16sheLL-containing samples in aqueous solution showed two distinct peaks, including one near 4,000 nm that corresponded to the sizes of mega-HPV L1 assemblies observed on unwashed p16sheLL VLP-loaded TEM grids. These large assemblies appeared capable of maintaining recognizable higher-order structural patterning despite variation in total particle size, consistent with the preservation of icosahedral assembly features. The following figures also include L1 antibody staining with 10 nm gold-conjugated particles, which were highly concentrated on these aggregates, as reflected by the increased staining intensity.

**FIGURE 30:**
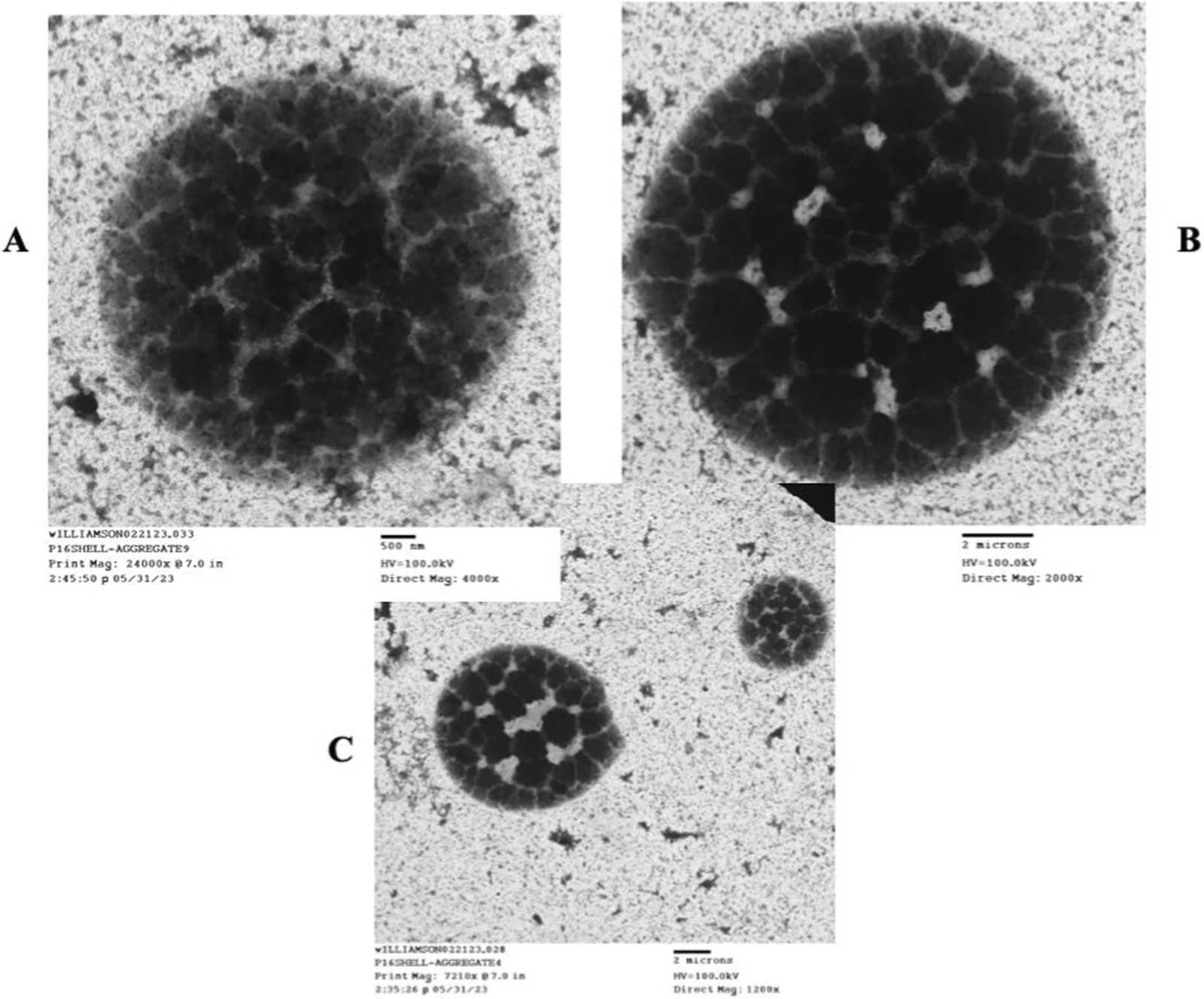
Electron micrographs of p16sheLL VLP aggregates in unwashed TEM samples. **(A)** Shows one mega aggregate with close detail of L1 mega-pentamer formation at a magnification of 4000X. **(B)** Shows an even larger aggregate with an amazingly detailed view of some distortions to the normal star shape at only ×2000 magnification. **(C)** Shows two megacapsids of different sizes at even lower magnification (1200X).

**FIGURE 31:**
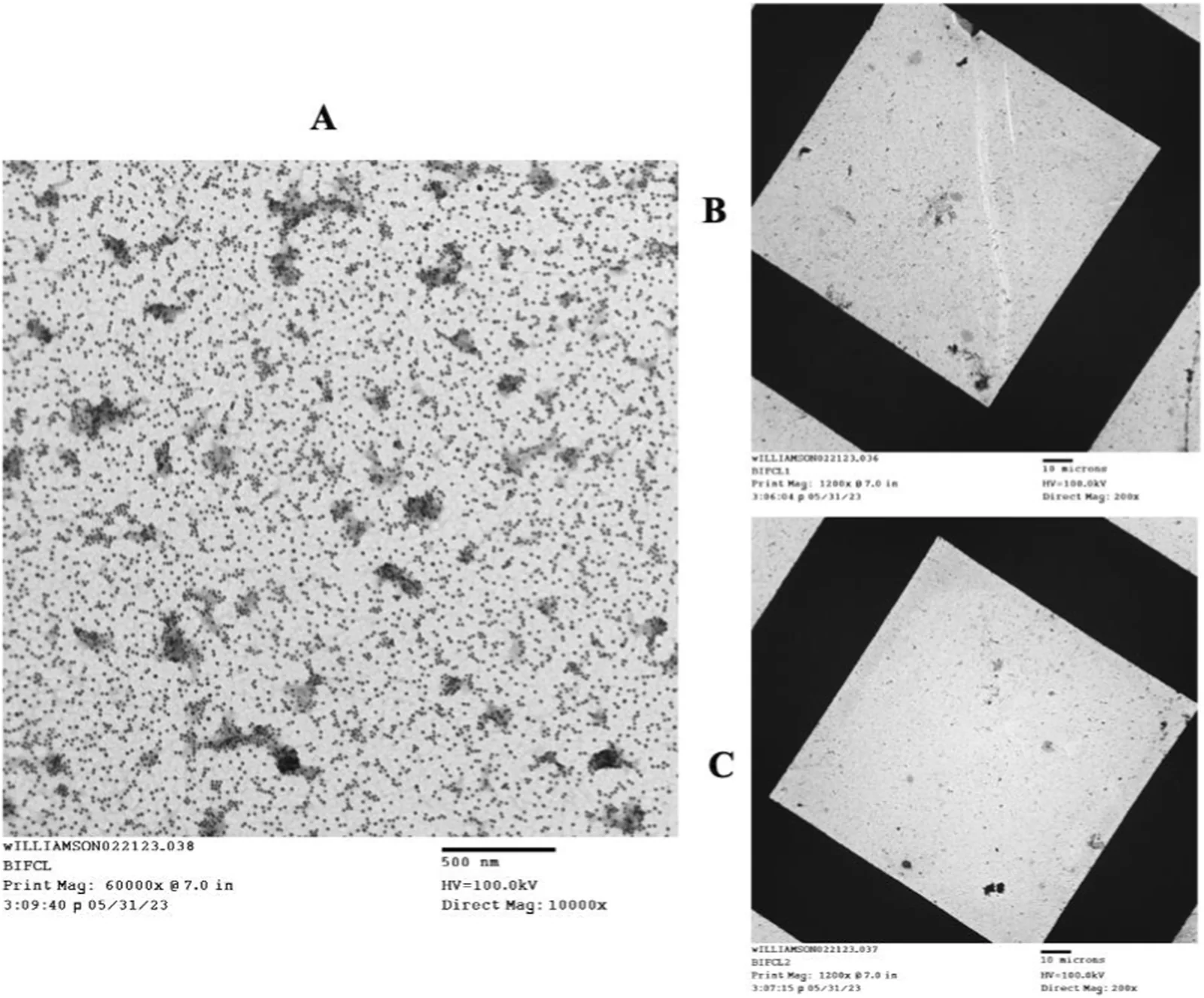
Electron micrographs of BiFC-VLPs in unwashed TEM samples. **(A)** is a close-up of the field, where VLPs are indicated as darkly stained, roughly circular shapes throughout the image. **(B,C)** Show the lowest magnification possible (200X) of unwashed grids containing BiFC-VLPs. The two boxes in **(B,C)** Represent the top two squares with the largest areas of concentrated stain out of the total 150 squares on the grid.

It is possible that the distorted star-like appearance observed between L1 “mega-pentamers” resulted in part from sample processing and 2D flattening of a 3D particle. Although the mega-VLPs were broadly reminiscent of native structure, clear aberrations were still present, including greater spacing between mega-pentamers and formations in which some mega-pentamers appeared to be absent. The largest aggregate particle recorded across samples measured 10 microns in diameter, representing an approximately 1000-fold increase over the single particles measured in Sections 3.6 and 3.7.

Adhesion, aggregation, motility, protein folding, and overall survival of most viruses are controlled by hydrophobic and Van der Waals forces (Bormashenko et al., 2021). Viruses and nanoparticles that are larger than the atomic scale but remain submicron in at least one dimension occupy the mesoscale. For reference, the total molecular weight estimated from various calculations mentioned in Sections 2 and 3 fell within the natural range (10^6^ Da–10^9^ Da) for most viruses, and BiFC-fused VLPs had a median estimated weight of 2.5 × 10^8^ Da. Mesoscale particles are significantly affected by spontaneous fluctuations in the system’s equilibrium (Bormashenko et al., 2021), which is important for determining measurement methods and constraints. In Table 2 from Section 3, temperature was kept constant across all samples to reduce variation in the system’s immediate kinetic energy. Viruses are also known to remain stable in aqueous environments and to be strongly influenced by water-mediated surface interactions (Gassilloud and Gantzer, 2005). It is therefore plausible that the large mega-VLP aggregates observed in p16sheLL samples prepared on unwashed grids reflected the absence of a DI water wash step. The p16sheLL grids washed with DI water after sample application may have allowed especially large particles, which had greater surface area for hydration and interaction, to move more readily into the aqueous phase.

The absence of mega-VLPs in both the washed and unwashed BiFC-VLP preparations suggests that the addition of the HPPAPKED linker and/or Venus fluorophore may have slightly altered the electrostatic and hydrophobic interactions in a way that reduced particle aggregation. Small clusters ranging from 3–5 particles were still observed in both p16sheLL and BiFC TEM images, and correct capsid formation was evident in individual BiFC VLPs. The addition of Venus and, potentially, the linkage of the fluorophore, capsid binding, and assembly are only inhibited at high aggregate concentrations. Correct capsid formation of individual particles is still unhindered by fluorophore linkage to the capsid proteins.

Zetasizer forward-scatter data for BiFC-VLPs included several noisy samples with lower-quality polydispersion indices, which could initially suggest fluorescent light interference from intact capsids displaying BiFC signal. Because this construct is novel, it was difficult to generate a truly appropriate positive control for DLS. In addition, the 633 nm wavelength emitted and detected by the Zetasizer lies outside the detection range of the Venus/BiFC signal, making it unlikely that the fluorophore itself was the primary source of noise. A more likely explanation is that BiFC samples contained relatively few large or aggregated particles, which are the principal targets of forward-scatter detection (Markova et al., 2022). In the aqueous environment of Zetasizer cuvettes, BiFC VLPs exhibited very little aggregation, whereas under the dry, fixed conditions required for TEM, particles occasionally appeared as aggregates up to 300 nm in diameter. During TEM preparation, particles may have formed doublets, triplets, or quadruplets because of water loss, charge attraction to the pre-treated acidic grids, or other processing-related effects. Notably, no larger aggregates were observed in the BiFC group after TEM preparation. In contrast, p16sheLL VLPs appeared to aggregate under both aqueous and dry preparation conditions, suggesting reduced stability across these conditions and raising concerns for downstream vaccine manufacturing workflows.

### 4.3 Future directions with flow virometry, resonant mass measurement, and field flow techniques

#### 4.3.1 Weight- and buoyancy-based sorting potential

Mesoscale particles are most often screened using low-throughput mass spectrometry (throughput 10^4^). Flow cytometry and flow virometry rely on multiple physical principles to separate particles based on size, acoustic and hydrodynamic focusing, and fluorescent activation (Tabler and Tilton, 2024). FACS can increase throughput and selection potential to 10^9+^, limited primarily by the number of particles in the sample and the instrument’s resolution (Robinson et al., 2026). As manufacturers and emerging technologies move toward nanoscale and microscale applications, the effects of surface tension, hydrodynamic properties, and other small-scale microenvironmental forces become increasingly important on a per-particle basis. Applications such as flow virometry and resonant mass analysis of very small particles must account for factors such as flow pressure, buoyancy, surface tension, the flow medium, and other related factors (Tabler and Tilton, 2024).

Newer theories in flow engineering allow reducing the lower limit of detection by adding Resonance Mass Measurement (RMM). RMM is another validated tool (in addition to or in place of Zetasizer instruments) commonly used to measure quantity and size (Krueger et al., 2019). Particle buoyant mass is measured by the change in frequency of a resonator as particles interact with it. This measurement is based on Archimedes’ Principle, which describes the buoyant force on an object, accounting for hydrostatic pressure and the object’s surface tension. The addition of buoyancy separation to bacterial cell recombinants was successfully selected, with higher throughput efficiency than current flow cytometers (Schmitt et al., 2018). Subvisible cell phenotypes had different densities that were directly proportional to the formation of a reporter enzyme. These advances suggest that extremely small particles and small weight fluctuations can be appropriately gated, with consideration of Archimedes’ Principle.

The addition of BiFC components to L1 and L2 increases the total weight of L1/L2-containing capsids, producing a greater weight difference than that observed for L1-only particles or unmodified p16sheLL particles. A linear trend was observed in both modified and unmodified VLP total weight (kDa) across the minimum and maximum L2 composition range (Figure 32). Simple linear regression showed that both groups differed significantly from a slope of 0 (F = 204.5, DF = 1, 19, p < 0.0001), and that the slopes also differed significantly between groups (F (1,38) = 12.03, p = 0.0013). Within each group, rows were compared using a 2-way ANOVA (F (20,20) = 33). Bonferroni-adjusted p = 0.0024 indicated a significant difference in the weight of L1-only particles and particles with 360 L1 + 12 L2 molecules (minimum L1/L2 weight) in both groups. Although BiFC modification shifts the total weight profile of the VLP relative to the p16sheLL construct, the incremental difference per additional L2 molecule remains too small to support conventional size-based separation. Only the particles at either extreme (L1 only and L1 + 32 L2) are significantly different in weight enough to be detected by nano detection methods.

**FIGURE 32:**
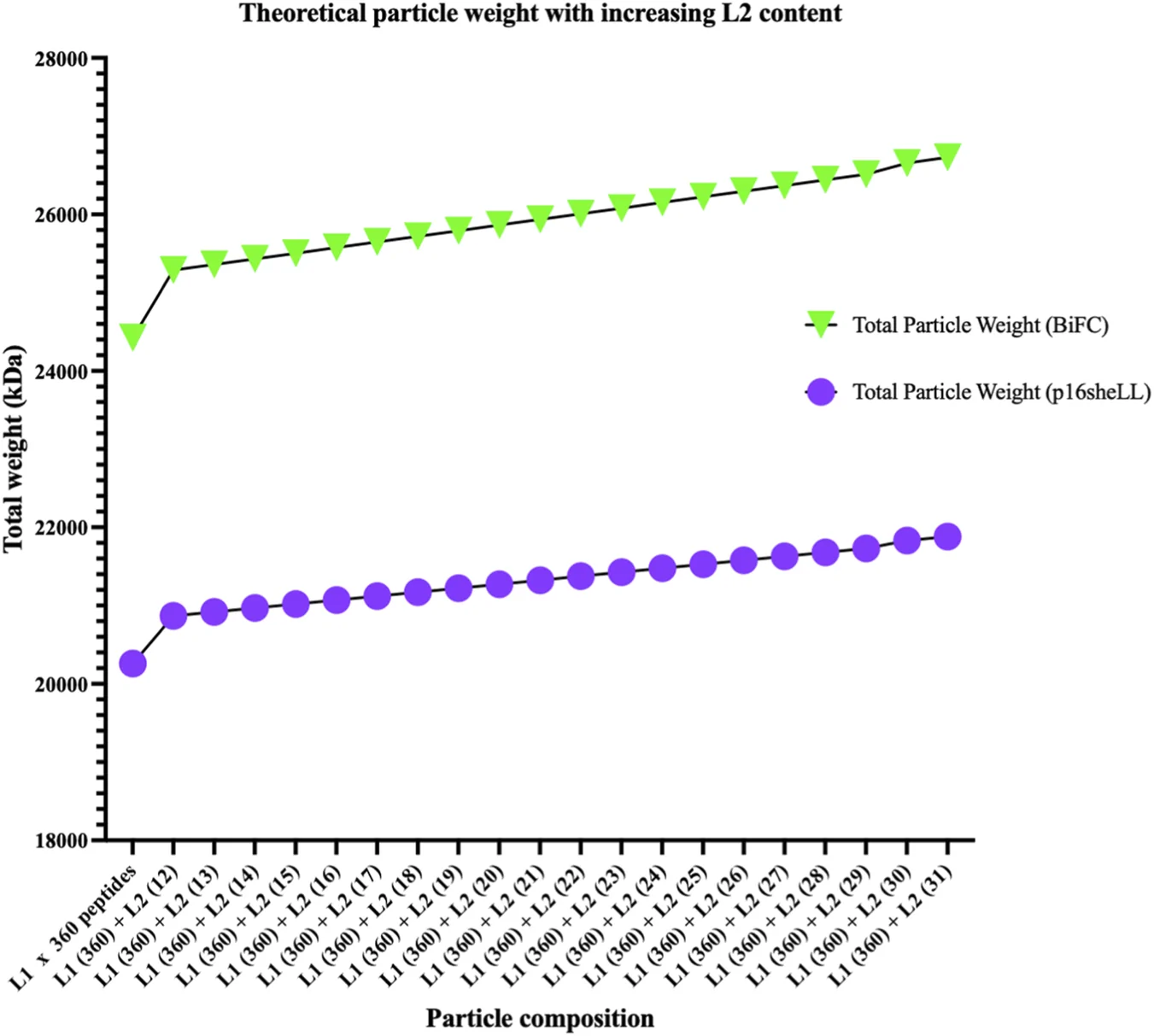
Total weight of HPV16 VLPs with and without BiFC modification, with increasing L2 content. The Green Triangles at the top represent the weights of BiFC-modified proteins. The purple circles represent the p16sheLL unmodified protein weights.

#### 4.3.2 Charge-based separation potential using field flow fractionation

Field-Flow Fractionation is another candidate for modifying current flow cytometer approaches for nanoparticle sorting (Giordani et al., 2023). A significant difference in charge was observed between L1/L2 BiFC VLPs and the other groups (L1-only BiFC VLPs or p16sheLL VLPs), which could enable separation of fully formed capsids based on charge using adapted liquid chromatography techniques. At physiological pH (7.3–7.4), the overall charge of L1 without BiFC is ∼ -3, whereas the BiFC-modified L1 protein is approximately +27. The L2 protein also shifts in overall charge from −6 to −9 with BiFC addition. The overall charge of the BiFC-modified fully formed capsids is much more positive compared to the native negatively charged particles, and L1/L2 is significantly less than L1-only charged particles in the BiFC-modified construct.

Current flow cytometer and field-flow fractionation instruments also share similar principles of sample uptake and laminar flow, making combined or adapted workflows conceptually plausible. Separation of particles based on charge and molecular weight could be achieved by adding a charged membrane and a crossflow step following initial sample injection, but before fluorescent detection through the optical cell. Under these conditions, charge differences between L1-only and L1/L2-containing particles could promote retention of one population while allowing the other to pass through first, generating temporal and spatial separation within the membrane-containing region of the flow path. Subsequent FACS-based analysis on an instrument capable of nanoparticle detection could then provide secondary confirmation of a fully functional, separated VLP population.

An additional advantage of integrating these approaches is that the environmental forces acting on mesoscale particles would remain more consistent across sorting conditions, potentially reducing variability introduced by differences in sample preparation and storage between purification and downstream analytical methods. In the context of HPV16 VLP formation, the hydrostatic modifications provided by BiFC conjugation are potentially different enough from specialized separation of VLPs containing both L1 and L2 on the basis of overall particle charge.

## 5 Conclusion

This project demonstrated the production of correctly formed HPV16-like capsids modified with a fluorescent BiFC tag, with the potential to allow for particle monitoring and sorting applications. Overall, the incorporation of BiFC enabled efficient detection of intracellular VLP production in producer cell populations across multiple excitation/emission filter combinations and imaging platforms while preserving capsid formation and reducing aggregation relative to similar constructs. The findings suggest that this construct could offer practical advantages in studies requiring accessible monitoring of VLP assembly, characterization of mixed particle populations, and improved recovery of usable particles from aqueous suspension.

If further studies confirm immunogenicity and *in vivo* functionality, the BiFC-VLPs could provide a useful platform for vaccine-oriented applications. In that regard, these particles could potentially be used to protect against tumor development in tissues with similar extracellular matrix components by priming the adaptive immune system, as seen in previously mentioned vaccine models. Combining BiFC-VLPs with a reported chimeric L2 epitope-presenting L1 protein (Slupetzky et al., 2007; Sanchooli et al., 2018) may provide a route toward broader cross-type protection in addition to type-specific protection associated with L1. Since VLPs were confirmed to be empty of genetic material, they may serve as a foundation for future gene and chemotherapeutic packaging for targeted delivery. The reduced aggregation of BiFC-modified particles further suggests that packaged therapies may be distributed more uniformly across targeted cell populations, while improving flow properties and reducing technical limitations associated with large aggregates. Additionally, the lack of aggregation should also facilitate a higher yield of usable viruses suspended in aqueous solutions (required for vaccine dosing, storage, and administration).

Although flow virometry was not available in this study, the combined use of TEM and Zetasizer analyses strongly suggests that this new construct may support more accurate measurement and future sorting of particle populations, with possible advantages in flow rate and reduced clogging in downstream applications. This work establishes and validates a novel HPV-16 VLP with a fluorophore attachment, preserving protein loop domains and structural flexibility while expanding the particle’s potential utility for monitoring, sorting, and future translational applications.

## Data Availability

The data supporting the conclusions of this article will be made available by the authors upon reasonable request.
